# Bedtime procrastination and psychological distress in university students: a systematic review and meta-analysis of their association

**DOI:** 10.3389/fpsyg.2026.1767938

**Published:** 2026-03-05

**Authors:** Obaid Azeem, Fatma Sulaiman, Zhu Haidong

**Affiliations:** Department of Applied Psychology, Normal College, Shihezi University, Shihezi, China

**Keywords:** anxiety, bedtime procrastination, depression, mental health, meta-analysis, sleep procrastination, stress, systematic review

## Abstract

Bedtime procrastination (BP), the voluntary delay of sleep despite the opportunity and intention to sleep, is prevalent among university students, a population particularly vulnerable to both sleep problems and psychological distress. While theoretically linked to mental health, the magnitude and consistency of its associations with depression, anxiety, and stress remain unclear. This systematic review and meta-analysis aimed to quantify these associations and explore potential moderators. We systematically searched PubMed, Web of Science, EBSCOhost, ScienceDirect, WanFang Data, and ProQuest from inception to 2025. Observational studies reporting correlations between BP and depression, anxiety, or perceived stress in university students were included. Study quality was assessed using the Joanna Briggs Institute checklists. Random-effects meta-analyses were conducted to synthesize Pearson correlation coefficients (*r*). Moderator analyses examined geographic region, gender, and measurement tool. Eighteen studies (*N* = 35,097) were included. Meta-analyses revealed significant, positive associations between BP and overall psychological distress (*k* = 14, *r* = 0.34, 95% CI [0.23, 0.43]), depressive symptoms (*k* = 11, *r* = 0.28, [0.22, 0.34]), anxiety symptoms (*k* = 8, *r* = 0.30, [0.22, 0.37]), and perceived stress (*k* = 9, *r* = 0.38, [0.30, 0.46]). Effect sizes were moderate, with the strongest association observed for stress. Substantial heterogeneity was present (*I^2^* > 90%). Moderator analyses indicated significantly stronger associations in samples from Asian regions compared to the non-Asian areas for overall distress and depression. Neither gender distribution nor the specific BP measurement tool significantly moderated the effects. Bedtime procrastination is a robust, transdiagnostic behavioral correlate of psychological distress in university students, showing a powerful link to perceived stress. Findings support theoretical models framing BP as a stress-contingent self-regulation failure. The association appears robust across methodological variations but is culturally contextualized. Targeting bedtime procrastination in interventions may offer a viable pathway for improving sleep and mental wellbeing in this population.

**Systematic review registration:**

https://osf.io/gy6pv/overview?view_only=cf344af529ec48a0a50e0ea09d6cb94d.

## 1 Introduction

University students frequently experience compromised sleep due to heightened academic and social stressors. While external demands are often implicated, a growing literature highlights an internal, behavioral factor: the voluntary delay of bedtime, or bedtime procrastination. Sleep represents a fundamental biological necessity, as critical to human functioning as nutrition and hydration. It is a complex, restorative process that underpins cognitive performance, emotional regulation, and physiological health (Walker, 2017). For university students, navigating a developmental period marked by significant academic demands, social transitions, and often newfound autonomy, obtaining sufficient and high-quality sleep is particularly vital. Adequate sleep forms the foundation for learning, memory consolidation, and academic achievement (Walker, 2017). Despite its importance, a substantial proportion of this population experiences chronic sleep deficiency and poor sleep quality, constituting a pressing public health concern within higher education globally (Lund et al., 2010).

University students represent a particularly vulnerable and informative population for investigating bedtime procrastination and its mental health associations for several reasons. First, this developmental period is characterized by newfound autonomy in sleep–wake schedules, reduced parental oversight, and high academic demands, creating a “perfect storm” for bedtime procrastination to emerge as a maladaptive coping strategy. Second, university students globally report high rates of both sleep deficiency and psychological distress, making this a pressing public health concern within higher education. Third, the university environment provides a relatively homogeneous context with shared stressors (academic pressure, social transitions, future uncertainty) and digital connectivity patterns, allowing for clearer examination of the BP-mental health relationship. Finally, identifying modifiable behavioral factors like bedtime procrastination in this population has direct implications for campus-based interventions and mental health promotion.

### 1.1 The problem of bedtime procrastination

While factors such as academic workload, social activities, and technology use are commonly implicated in student sleep loss, a more specific behavioral tendency has been identified: bedtime procrastination. Bedtime procrastination is defined as the volitional delay of going to bed, despite having the opportunity and intention to do so, without external causes (Kroese et al., 2014). It represents a domain-specific form of general procrastination, where individuals choose more immediately rewarding activities (e.g., social media use) over sleep (Exelmans and den Bulck, 2016).

The university environment, characterized by reduced parental oversight, flexible schedules, and constant digital connectivity, creates conditions ripe for bedtime procrastination to flourish. Furthermore, the daily cognitive and self-regulatory demands of student life can deplete mental resources by evening, impairing the capacity to resist immediate gratification and adhere to bedtime intentions (Kroese et al., 2016). This pattern can lead to a recurrent, intentional truncation of sleep opportunity, resulting in cumulative sleep debt.

### 1.2 Consequences of sleep loss and mental health links

The sequelae of chronic sleep insufficiency are extensive and especially detrimental for students. Cognitive domains essential for academic success, including attention, concentration, and memory, are significantly impaired by inadequate sleep (Diekelmann et al., 2009). Extensive evidence links sleep disturbances to mental health difficulties through complex, often bidirectional relationships. Insomnia is both a core symptom and a significant predictive correlate of the onset of major depressive disorder (Baglioni et al., 2011). Similarly, anxiety disorders are highly comorbid with sleep problems, where pre-sleep cognitive arousal hinders sleep onset, and sleep loss, in turn, exacerbates emotional reactivity and anxiety sensitivity (Alfano et al., 2009). Stress, a ubiquitous feature of student life, is also amplified by poor sleep; sleep deprivation heightens physiological and subjective responses to subsequent stressors (Yoo et al., 2007).

### 1.3 Existing syntheses and the need for the current review

Previous systematic reviews and meta-analyses have examined correlates of bedtime procrastination, with a primary focus on sleep outcomes. For instance, Hill et al. (2022) conducted a comprehensive meta-analysis identifying consistent associations between bedtime procrastination and poorer sleep quality, shorter sleep duration, and later sleep timing. While their synthesis included mental health variables as secondary correlates, it did not quantitatively aggregate the strength of relationships between bedtime procrastination and specific mental health outcomes—depression, anxiety, and perceived stress—within the university student population. Other narrative reviews have discussed bedtime procrastination as a potential behavioral correlate for psychological distress, recent advances in intensive longitudinal methods (Schmiedek et al., 2023) offer opportunities to examine within-person processes linking daily stress to bedtime procrastination, but have not provided pooled effect estimates or examined moderators of these associations. Consequently, the magnitude, consistency, and potential contextual factors influencing the relationship between bedtime procrastination and mental health in university students remain unclear. The present systematic review and meta-analysis directly addresses this gap by: (1) providing the first quantitative synthesis of correlations between bedtime procrastination and depression, anxiety, and stress in university students; (2) examining key moderators (e.g., geographic region, measurement tools) to explain heterogeneity; and (3) integrating findings within a theoretical framework that positions bedtime procrastination as a stress-contingent self-regulation failure. This focused synthesis is crucial for informing targeted interventions in a population experiencing high rates of both sleep problems and psychological distress.

### 1.4 Bridging the gap: bedtime procrastination as a potential behavioral correlate

Whereas traditional insomnia often involves an inability to sleep despite the opportunity, bedtime procrastination represents a distinct pathway to sleep deficiency—one driven by a failure to disengage from waking activities. This pathway, culminating in insufficient sleep, may therefore constitute a modifiable behavioral correlate of psychological distress. Preliminary research supports this proposition, demonstrating moderate-to-strong correlations between higher bedtime procrastination and elevated symptoms of depression, anxiety, and perceived stress among university students (Kroese et al., 2014; Sirois et al., 2019). This suggests a distinct etiological pathway: a behavioral choice (procrastination) leads to sleep deficiency. This deficiency may then confer risk for psychological distress through direct neurobiological effects on emotional regulation and indirect effects via daytime functional impairment (Walker, 2009).

### 1.5 Rationale for the present review

However, the literature examining these specific relationships remains fragmented. Inconsistencies in reported effect sizes may stem from variations in methodological quality, sample characteristics, and measurement tools. Consequently, a quantitative synthesis is lacking, and the overall strength and consistency of these associations remain unclear. A systematic review and meta-analysis are therefore necessary to consolidate empirical findings, quantify the overall strength of these associations, assess the quality of extant research, and identify potential moderators. Clarifying these relationships is crucial for translating research into practice.

Therefore, a systematic review and meta-analysis are necessary to: (1) quantify the overall strength of these associations, (2) assess methodological quality and identify sources of heterogeneity, and (3) integrate findings into a cohesive theoretical model. This synthesis will establish whether BP represents a consistent and modifiable behavioral correlate for interventions aimed at mitigating the student mental health crisis.

## 2 Theoretical framework

This review is grounded in an integrative theoretical framework positing that bedtime procrastination contributes to depression, anxiety, and stress in university students primarily through the pathway of volitional sleep reduction. The model synthesizes key tenets from self-regulation theory, sleep science, and established models of psychopathology.

### 2.1 Bedtime procrastination as a self-regulation failure

The core behavior is defined as a voluntary delay of bedtime despite the opportunity and intention to sleep, absent external impediments (Kroese et al., 2014). Conceptually, bedtime procrastination is best understood as a failure of self-regulation (Kroese et al., 2016), which involves the capacity to align one’s actions with long-term goals by managing impulses, emotions, and attention. The high cognitive and self-regulatory demands of university life can deplete these finite mental resources by day’s end, a state known as ego depletion (Baumeister et al., 1998). In this depleted state, the immediate gratification offered by digital media or other leisure activities disproportionately outweighs the abstract, long-term value of sufficient sleep, leading to a failure to enact the bedtime intention (Nauts et al., 2019).

### 2.2 The consequence: sleep insufficiency and circadian disruption

The direct outcome of habitual bedtime procrastination is sleep insufficiency—a chronic deficit in total sleep time relative to one’s physiological need (Alotaibi et al., 2020). Furthermore, inconsistent bedtimes disrupt the body’s endogenous circadian rhythms (Zisberg et al., 2010). This misalignment impairs sleep quality and stability, creating a compounded state of sleep deficiency that affects neurobehavioral function.

### 2.3 Pathways to negative mental health outcomes

The established links between poor sleep and worsened mental health provide the critical bridging mechanisms in this framework. Specifically, we propose that sleep insufficiency stemming from BP influences mental health through (1) cognitive, (2) neurobiological, and (3) physiological stress pathways.

Cognitive pathway: The Cognitive Model of Insomnia (Harvey, 2002) posits that sleep-related anxiety and catastrophizing about consequences form a vicious cycle. A student who procrastinates on sleep may experience increased pre-sleep cognitive arousal (“I’ll be exhausted tomorrow”), which further delays sleep onset and fuels next-day anxiety and rumination.

Neurobiological pathway: Sleep, particularly rapid-eye-movement (REM) sleep, is essential for the overnight processing and regulation of emotional memories (Walker, 2009). Chronic sleep deprivation weakens top-down control from the prefrontal cortex while enhancing bottom-up reactivity of the amygdala. This neural profile predisposes individuals to heightened emotional reactivity, reduced impulse control, and a negative affective bias: core features of anxiety and depression.

Physiological stress pathway: Sleep serves as a key period for the down-regulation of the hypothalamic–pituitary–adrenal (HPA) axis. Sleep loss dysregulates this system, leading to elevated and prolonged cortisol secretion (Zhang et al., 2024). This creates a persistent physiological state of heightened stress, lowering the threshold for perceiving and reacting to daily academic and social stressors with greater intensity.

### 2.4 Synthesized model

In summary, this framework proposes a sequential model:

1. Bedtime Procrastination emerges from a state of depleted self-regulatory capacity.
2. This behavior directly causes Sleep Insufficiency and Circadian Disruption.
3. The resultant poor sleep then propagates risk for Depression, Anxiety, and Stress via synergistic cognitive (maladaptive thoughts), neurobiological (impaired emotional regulation), and physiological (HPA-axis dysregulation) mechanisms.

This integrated model frames bedtime procrastination as a modifiable behavioral entry point into a cycle of sleep loss and deteriorating mental health. This cycle is mediated through synergistic cognitive, neurobiological, and physiological mechanisms.

**Figure 1:**
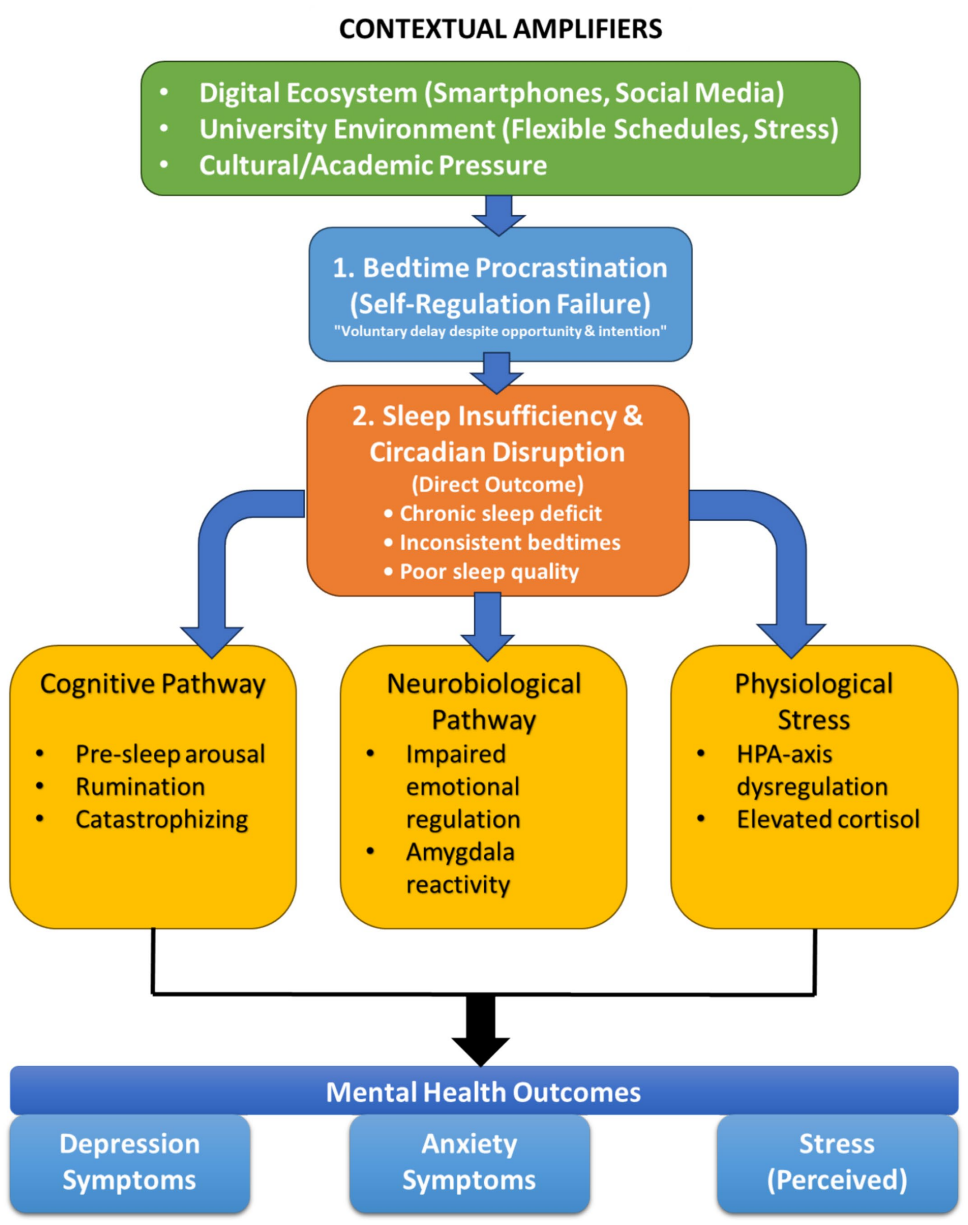
Conceptual model linking bedtime procrastination to mental health outcomes via sleep insufficiency and three mediating pathways (cognitive, neurobiological, and physiological) in university students.

This integrated model, summarized visually in Figure 1, frames bedtime procrastination as a modifiable behavioral entry point into a cycle of sleep loss and deteriorating mental health. The model illustrates how bedtime procrastination, stemming from depleted self-regulation in the context of digital and academic pressures, leads to sleep insufficiency and circadian disruption. These sleep deficits then propagate risk for depression, anxiety, and stress through cognitive (e.g., pre-sleep arousal), neurobiological (e.g., impaired emotional regulation), and physiological (e.g., HPA-axis dysregulation) pathways.

## 3 Methods

This systematic review and meta-analysis were conducted and reported in accordance with the Preferred Reporting Items for Systematic Reviews and Meta-Analyses (PRISMA) 2020 statement (Page et al., 2021).

### 3.1 Research question

Is bedtime procrastination significantly associated with higher levels of depression, anxiety, and stress in university students?

### 3.2 Research objectives

The primary aim of this systematic review and meta-analysis is to synthesize the evidence on the association between bedtime procrastination and symptoms of depression, anxiety, and stress in university students. The specific objectives are to:

1. Systematically identify and select observational studies that examine bedtime procrastination and mental health outcomes in university student populations.
2. Quantify the overall pooled association between bedtime procrastination and general psychological distress (composite of depression, anxiety, and stress).
3. Quantify the specific pooled associations between bedtime procrastination and:
  - Depressive symptoms.
  - Anxiety symptoms.
  - Perceived stress.
4. Investigate potential moderators of these associations, including geographic region, sample gender distribution, and the specific measure of bedtime procrastination employed.
5. Methodologically appraise the quality and risk of bias of the included studies.
6. Summarize and evaluate the evidence regarding sleep insufficiency as a proposed mechanistic pathway linking bedtime procrastination to adverse mental health outcomes.

### 3.3 Eligibility criteria (PICO framework)

The review question was structured using the PICO framework to define the scope of the search and inclusion.

- Population (P): University/college students (undergraduates and postgraduates). Excluded: High school students, vocational/training students not in university settings, and general adult populations.
- Exposure (I): Bedtime procrastination or sleep procrastination.
- Comparison (C): Lower levels or absence of bedtime procrastination.
- Outcomes (O): Symptoms of depression, anxiety, or perceived stress.

#### 3.3.1 Inclusion and exclusion criteria

Based on the PICO framework, the following specific criteria were applied.

##### 3.3.1.1 Inclusion criteria

Studies were included if they met all of the following criteria:

1. Study Design: Observational studies (cross-sectional, cohort, or case–control).
2. Participants: The sample consisted entirely or primarily (i.e., >80%) of university/college students or undergraduates.
3. Variables: The study quantitatively assessed the relationship between bedtime procrastination (or sleep procrastination) and at least one of the mental health outcomes (depression, anxiety, or perceived stress).
4. Publication Type: Original research published in a peer-reviewed journal.
5. Language: Published in English.

##### 3.3.1.2 Exclusion criteria

Studies were excluded based on any of the following:

1. Study Design: Qualitative studies, narrative or systematic reviews, meta-analyses, editorials, commentaries, conference abstracts, theses, and dissertations.
2. Population: Studies where the sample does not primarily consist of university/college students (e.g., high school students, general adults, clinical samples) or where university students constituted <80% of the sample.
3. Relevance: Studies that measured general procrastination but not *bedtime procrastination* specifically, or studies that measured mental health but did not analyze its relationship with bedtime procrastination.
4. Data: Full text not available or insufficient data reported to calculate or determine an effect size (e.g., correlation coefficient, regression coefficient).

#### 3.3.2 Information sources and search strategy

A systematic search was conducted across six electronic databases from inception until August 2025, with no language or date restrictions applied: PubMed, Web of Science, EBSCOhost (including APA PsycINFO), ScienceDirect, WanFang Data, and ProQuest Dissertations and Theses Global.

Our search included WanFang Data to capture relevant research published in English by Chinese scholars, as this database indexes both Chinese and English-language publications from China. We did not include CNKI (China National Knowledge Infrastructure) because: (1) CNKI primarily indexes Chinese-language publications, and our inclusion criteria were restricted to English-language publications to ensure consistent interpretation and quality assessment by the review team; (2) WanFang Data provides substantial coverage of English-language research from Chinese institutions relevant to our topic. While this approach may have excluded some Chinese-language studies, it ensured methodological rigor in screening and data extraction, given the review team’s language capabilities. The decision reflects a balance between comprehensiveness and practical constraints in conducting a systematic review.

The search strategy was designed to capture the PICO concepts. The following search string was adapted for each database:

(“bedtime procrastination” OR “sleep procrastination”) AND (“university student” OR “college student” OR undergraduate) AND (depress OR anxi* OR stress)

For WanFang Data, we employed the English search string as shown, as this database indexes English-language publications from Chinese institutions. While this approach may have limited the retrieval of Chinese-only studies, it aligned with our inclusion criteria, restricting to English-language publications.

Truncation symbols (*) were used to include related terms. The initial search yielded 480 records. Database-specific results are presented in Table 1.

**Table 1:** Database search results.

S. no.	Database	Initial hits	After filters	For title screening
1	PubMed	19	18	17
2	Web of Science	41	41	37
3	EBSCOhost	11	11	7
4	ScienceDirect	70	53	24
5	WanFang	24	23	23
6	ProQuest	315	246	52
Total		480	392	160

#### 3.3.3 Study selection process

**Figure 2:**
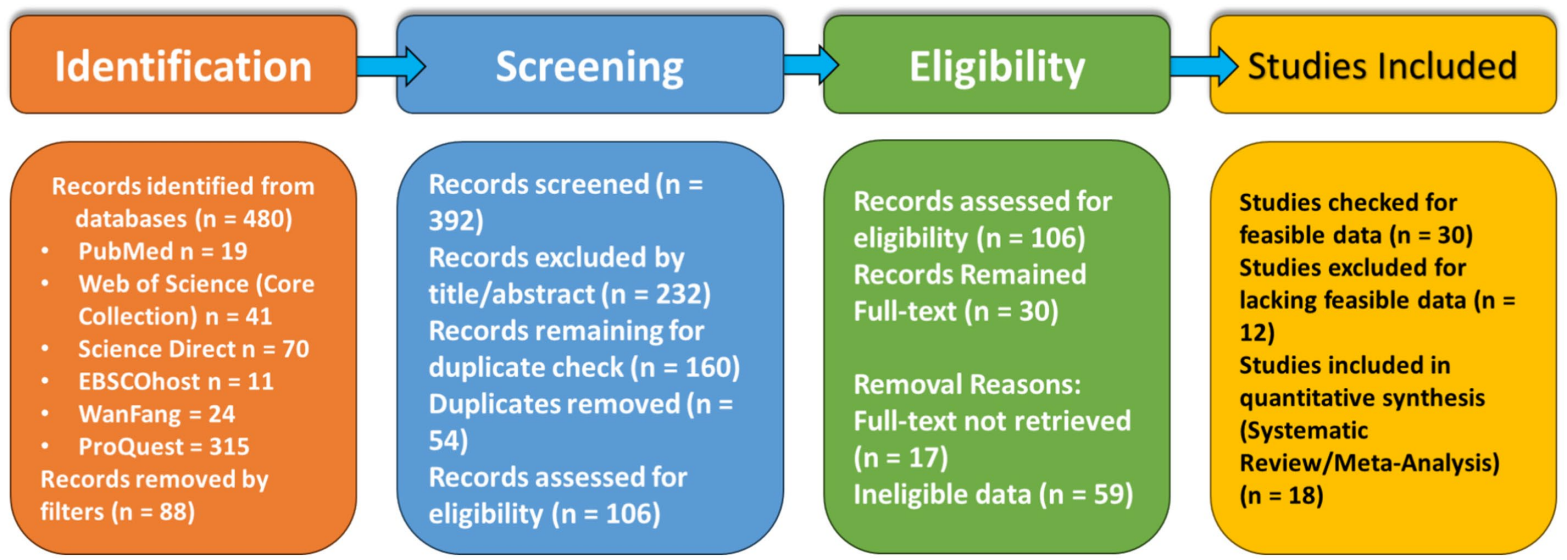
PRISMA (Preferred Reporting Items for Systematic Reviews and Meta-Analyses) flow diagram illustrating the study selection process for the systematic review. The diagram details the number of records identified, screened, assessed for eligibility, and included in the review, along with reasons for exclusions at each stage. The systematic search was conducted across six electronic databases (PubMed, Web of Science, EBSCOhost, ScienceDirect, WanFang, and ProQuest) using a predefined search strategy. Initial filters pertained to publication date, language, and study type. Articles were excluded during title screening for clear irrelevance to the research question and during full-text assessment for not meeting specific population, intervention, comparator, or outcome criteria, or due to inaccessible data or duplicate reporting.

The selection process was managed using Zotero reference manager (v6.0.30) and followed the stages detailed in the PRISMA flow diagram (Figure 2):

1. Initial Identification and Filtering: 480 records were identified. Basic filters (publication type, language) removed 88 records, leaving 392.
2. Title Screening: Titles of 392 records were screened for clear irrelevance, resulting in 160 records retained for abstract review.
3. Duplicate Screening: 54 duplicates were removed, leaving 106 unique records.
4. Full-Text Retrieval and Screening: Full texts were retrieved for 89 of the 106 records (17 were inaccessible). These 89 articles underwent deep screening against the eligibility criteria above, excluding 59.
5. Final Inclusion: The remaining 30 articles were assessed for feasible data relevant to the research question. Twelve were excluded, resulting in 18 studies included for synthesis and meta-analysis.

### 3.4 Data extraction

A standardized data extraction form was created in Microsoft Excel (v2405). The extracted dataset, including study characteristics and effect sizes, is provided in the Supplementary Dataset. One reviewer extracted data, which was verified by a second. Extracted data included:

- Study characteristics (author, year, country, design).
- Sample characteristics (N, mean age, gender distribution).
- Measurement tools for bedtime procrastination and mental health outcomes, with reported reliability (e.g., Cronbach’s *α*).
- Statistical data for the association between variables (correlation coefficients, regression coefficients, means/standard deviations).

### 3.5 Risk of bias assessment

The methodological quality of included studies was assessed independently by two reviewers using the Joanna Briggs Institute (JBI) critical appraisal checklists (Joanna Briggs Institute, 2017, 2020), with specific tools applied based on study design. This tool evaluates critical domains such as sample representativeness, exposure and outcome measurement, control for confounding, and statistical integrity. Disagreements were resolved by consensus.

### 3.6 Data synthesis and analysis

All meta-analytic procedures were performed using Comprehensive Meta-Analysis software (Version 4.0; Biostat Inc., Englewood, NJ, USA). Descriptive statistics and data preparation were conducted in IBM SPSS Statistics (Version 29.0.1.0). The authors used DeepSeek (version 2025) by DeepSeek AI^1^ to assist with language editing, formatting suggestions, and improving the clarity of certain passages in the manuscript. All AI-generated content was reviewed, fact-checked, and revised by the authors, who take full responsibility for the final content.

Effect Size Calculation: The primary effect size was the Pearson correlation coefficient (r). Where studies reported alternative statistics (e.g., standardized beta coefficients, odds ratios), these were converted to r using established formulas (Borenstein et al., 2009). Specifically, odds ratios were converted to correlation coefficients using the method described by Chinn (2000). All r values were transformed to Fisher’s z for analysis to stabilize variance, with final results back-transformed to r for interpretation.

Meta-Analytic Model: A random-effects model was selected *a priori*, as we assumed the true effect would vary across studies due to methodological and population differences.

Primary Analyses: Four distinct meta-analyses were conducted:

1. Overall association between bedtime procrastination and general psychological distress (a composite of depression, anxiety, and stress scores).
2. Specific associations between bedtime procrastination and (a) depressive symptoms, (b) anxiety symptoms, and (c) perceived stress.

Heterogeneity Assessment: Statistical heterogeneity was quantified using Cochran’s *Q* test (significance set at *p* < 0.10) and the *I^2^* statistic. *I^2^* values were interpreted as: 0–40% (low heterogeneity), 30–60% (moderate heterogeneity), 50–90% (substantial heterogeneity), and 75–100% (considerable heterogeneity).

Moderator Analysis: For meta-analyses with ≥10 studies (k), mixed-effects subgroup analyses and univariate meta-regressions were conducted to examine whether geographic region (Asia vs. non-Asia), gender composition (% female), bedtime procrastination measurement tool (BPS-9 vs. other), or study quality (based on Joanna Briggs Institute critical appraisal scores) moderated the observed associations.

Publication Bias Assessment: For meta-analyses with ≥10 studies, potential publication bias was assessed via: (a) visual inspection of funnel plots (effect size vs. standard error), (b) Egger’s weighted regression test, and (c) the trim-and-fill method to estimate and adjust for potentially missing studies.

Sensitivity Analyses: To assess robustness, leave-one-out sensitivity analyses were conducted by iteratively removing each study and recalculating the pooled effect size. Consistency between random- and fixed-effects models was also examined.

Risk of Bias Synthesis: Results from the Joanna Briggs Institute critical appraisal checklists were synthesized descriptively and visualized using the Robvis web tool.

## 4 Results

### 4.1 Study selection and characteristics

This meta-analysis of 18 studies and over 35,000 university students suggests bedtime procrastination (BP) as a significant, transdiagnostic correlate of psychological distress, though the predominantly cross-sectional nature of the evidence requires cautious interpretation of directionality and causality. A total of 18 studies (*N* = 35,097 university students) met the eligibility criteria for inclusion in the systematic review. The selection process is detailed in the PRISMA flow diagram (Figure 2). The majority of studies were cross-sectional (83.3%) and conducted in China (72.2%). The Bedtime Procrastination Scale (BPS-9) was the most common measure of BP (77.8% of studies). Mental health outcomes were assessed using validated instruments, including the Depression, Anxiety, and Stress Scales (DASS-21), Beck Depression Inventory (BDI-II), and Perceived Stress Scale (PSS). A complete overview of study characteristics is provided in Supplementary Table S1. While Cong et al. (2024) met inclusion criteria for the systematic review, it was excluded from the overall meta-analysis due to insufficient effect size data; however, its findings on academic stress and bedtime procrastination are discussed in the qualitative synthesis.

### 4.2 Meta-analytic results: associations between bedtime procrastination and mental health outcomes

Four separate random-effects meta-analyses were conducted to quantify the associations between bedtime procrastination and overall psychological distress, depressive symptoms, anxiety symptoms, and perceived stress. The main results are summarized in Table 2.

**Table 2:** Summary of random-effects meta-analyses.

Outcome	*k*	Total *N*	Pooled *r* [95% CI]	*p*-value	*I^2^* (%)	τ^2^
Overall psychological distress	14	20,450	0.336 [0.232, 0.432]	<0.001	97.9	0.044
Depressive symptoms	11	15,218	0.277 [0.218, 0.335]	<0.001	94.7	0.012
Anxiety symptoms	8	19,319	0.295 [0.221, 0.367]	<0.001	95.5	0.014
Perceived stress	9	12,582	0.383 [0.298, 0.463]	<0.001	98.4	0.027

*k* = number of studies; CI = confidence interval; *I*^2^ = heterogeneity index; τ^2^ = between-study variance.

Overall Pattern: Bedtime procrastination demonstrated significant, positive associations with all mental health outcomes (*p* < 0.001 for all). Effect sizes ranged from small to moderate for depression (*r* = 0.28) and anxiety (*r* = 0.30) to moderate for perceived stress (*r* = 0.38) and overall distress (*r* = 0.34). The strongest association was observed with perceived stress.

Heterogeneity: Substantial and statistically significant heterogeneity was present across all meta-analyses (*I^2^* > 90%), indicating considerable variability in effect sizes across studies. This heterogeneity warranted further investigation via moderator and sensitivity analyses.

**Figure 3:**
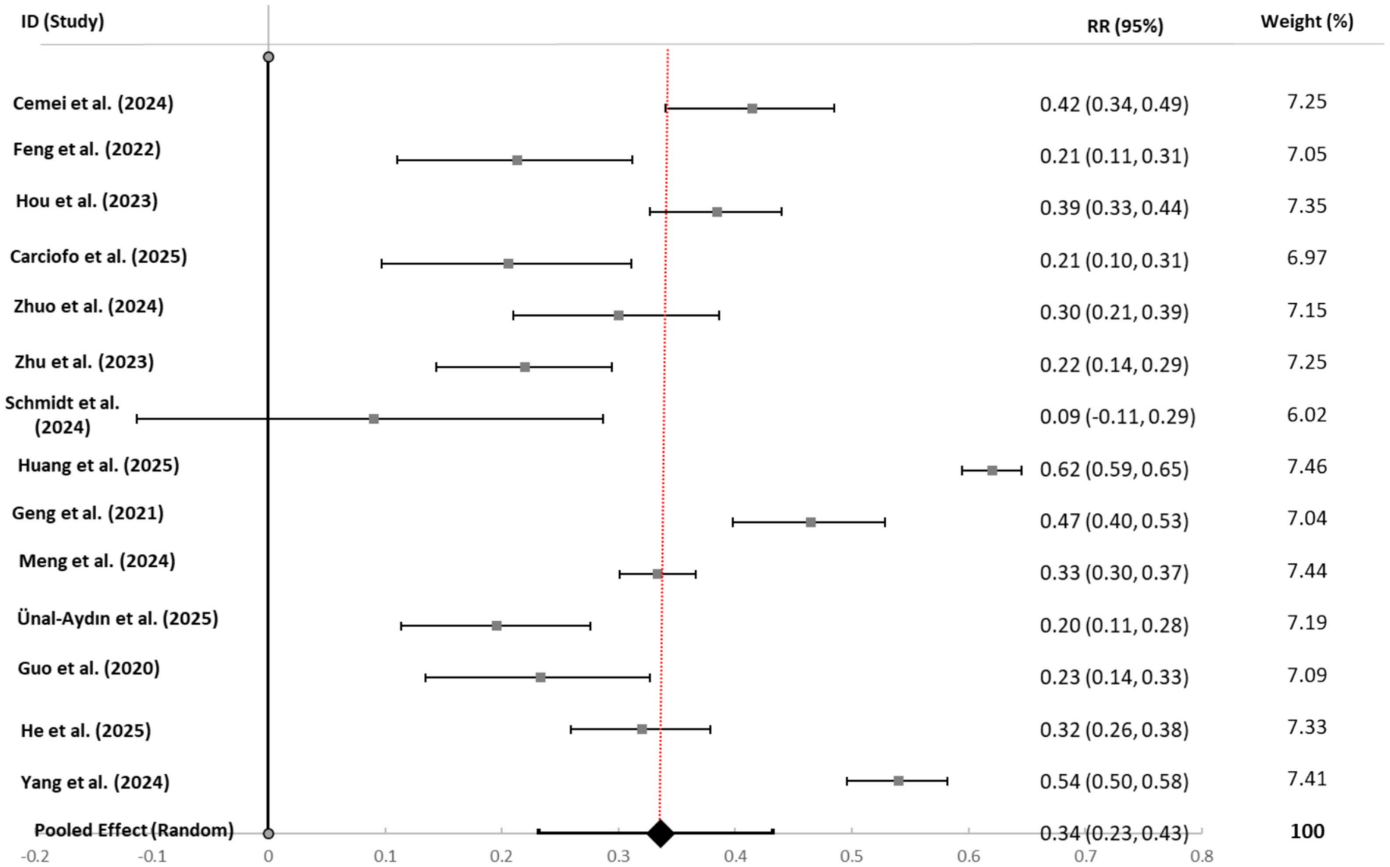
Forest plot of correlation coefficients between bedtime procrastination and overall psychological distress. Gray squares represent individual study effect sizes; horizontal lines indicate 95% confidence intervals; the black diamond represents the pooled random-effects estimate. The solid black vertical line at *r* = 0 indicates the null value (no association). The red dashed vertical line facilitates visual comparison with individual studies. Heterogeneity: *I*^2^ = 97.9%, τ^2^ = 0.044, *p* < 0.001.

**Figure 4:**
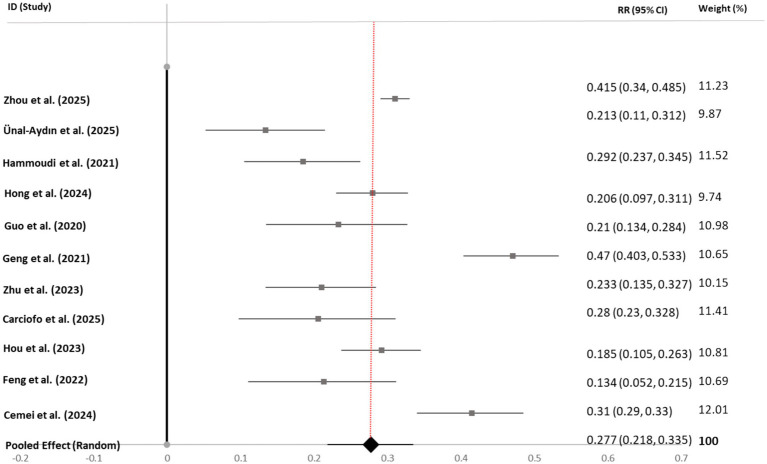
Forest plot of correlation coefficients between bedtime procrastination and depressive symptoms. Gray squares represent individual study effect sizes; horizontal lines indicate 95% confidence intervals; the black diamond represents the pooled random-effects estimate. The solid black vertical line at *r* = 0 indicates the null value (no association). The red dashed vertical line facilitates visual comparison with individual studies. Heterogeneity: *I*^2^ = 94.7%, τ^2^ = 0.012, *p* < 0.001.

**Figure 5:**
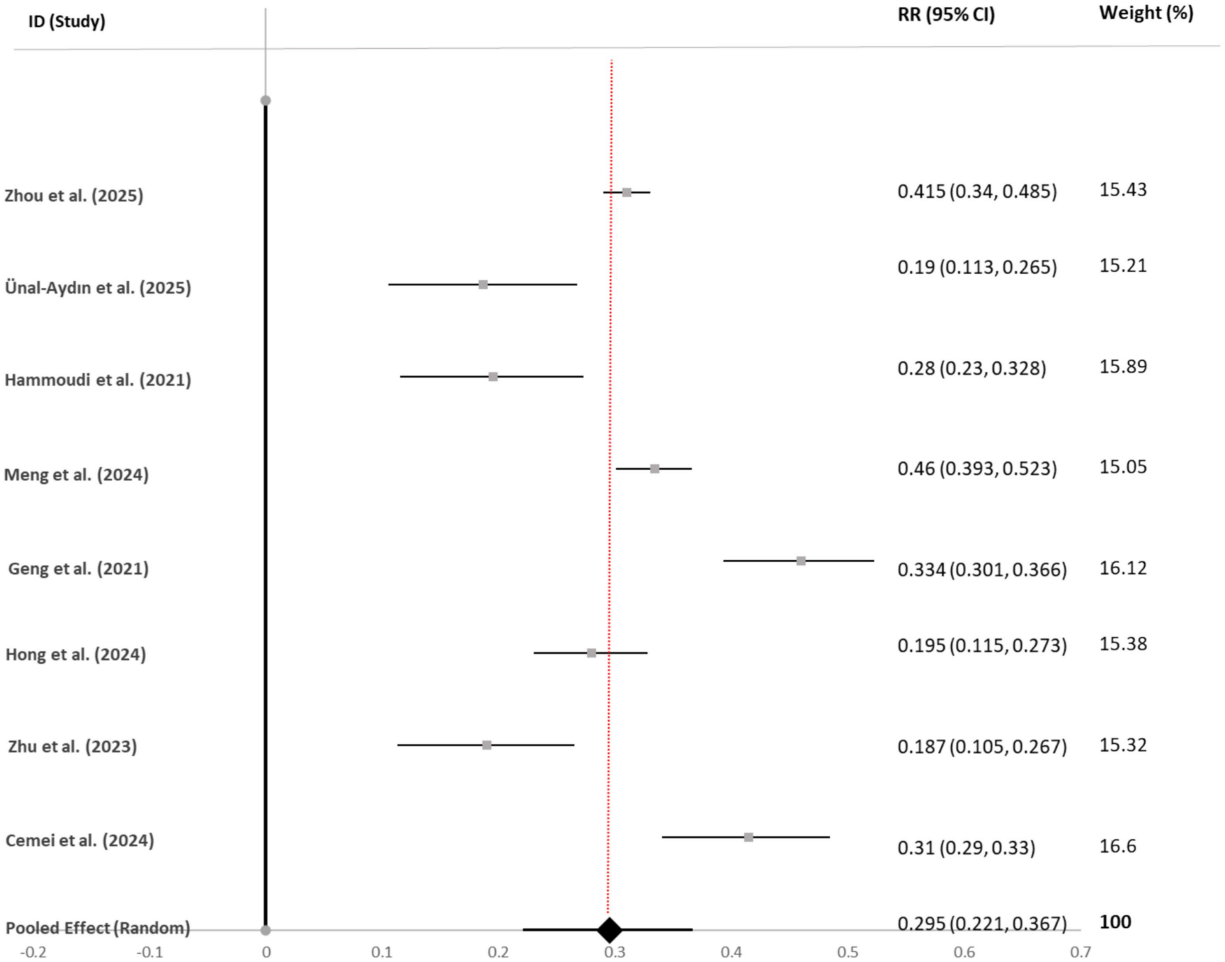
Forest plot of correlation coefficients between bedtime procrastination and anxiety symptoms. Gray squares represent individual study effect sizes; horizontal lines indicate 95% confidence intervals; the black diamond represents the pooled random-effects estimate. The solid black vertical line at *r* = 0 indicates the null value (no association). The red dashed vertical line facilitates visual comparison with individual studies. Heterogeneity: *I*^2^ = 95.5%, τ^2^ = 0.014, *p* < 0.001.

**Figure 6:**
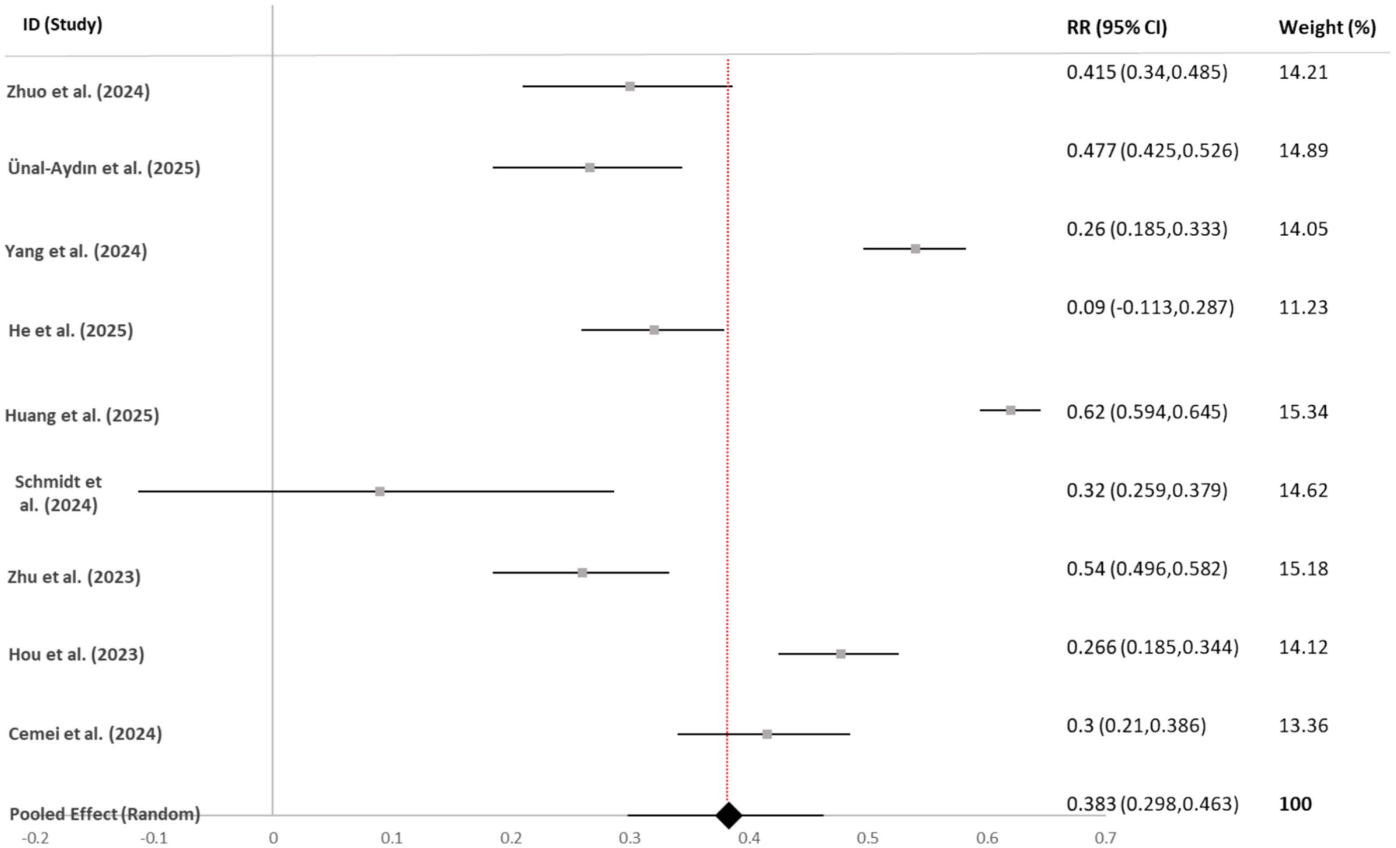
Forest plot of correlation coefficients between bedtime procrastination and perceived stress. Gray squares represent individual study effect sizes; horizontal lines indicate 95% confidence intervals; the black diamond represents the pooled random-effects estimate. The solid black vertical line at *r* = 0 indicates the null value (no association). The red dashed vertical line facilitates visual comparison with individual studies. Heterogeneity: *I*^2^ = 98.4%, τ^2^ = 0.027, *p* < 0.001.

Visual Synthesis: Forest plots for each outcome (see Figures 3–6) consistently showed positive associations. While most individual study effects were significant, a few studies—notably Schmidt et al. (2024)—reported weaker, non-significant correlations, contributing to the observed heterogeneity.

### 4.3 Detailed results by outcome

#### 4.3.1 Overall psychological distress

The meta-analysis of 14 studies (*N* = 20,450) revealed a pooled correlation of r = 0.34 (95% CI [0.23, 0.43]). All but one study reported significant positive correlations. The funnel plot showed slight asymmetry, and Egger’s test was significant (*p* = 0.008), suggesting potential publication bias. The trim-and-fill adjustment imputed two studies, yielding a slightly reduced but still significant effect (*r* = 0.30 [0.20, 0.40]). A leave-one-out sensitivity analysis confirmed the robustness of the finding (range: *r* = 0.32 to 0.35). Study-specific correlations are detailed in Table 3.

**Table 3:** Study-specific and pooled correlation coefficients for bedtime procrastination and overall psychological distress.

Study	*r*	95% CI	Weight (%)
Cemei et al. (2024)	0.415	[0.340, 0.485]	7.25
Feng et al. (2022)	0.213	[0.110, 0.312]	7.05
Hou and Hu (2023)	0.385	[0.327, 0.440]	7.35
Carciofo and Cheung (2025)	0.206	[0.097, 0.311]	6.97
Zhuo (2024)	0.300	[0.210, 0.386]	7.15
Zhu et al. (2023)	0.220	[0.144, 0.294]	7.25
Schmidt et al. (2024)	0.090	[−0.113, 0.287]	6.02
Huang et al. (2025)	0.620	[0.594, 0.645]	7.46
Geng et al. (2021)	0.465	[0.398, 0.528]	7.04
Meng et al. (2024)	0.334	[0.301, 0.366]	7.44
Aydın (2025)	0.196	[0.114, 0.276]	7.19
Guo et al. (2020)	0.233	[0.135, 0.327]	7.09
He et al. (2025)	0.320	[0.259, 0.379]	7.33
Yang et al. (2024)	0.540	[0.496, 0.582]	7.41
Pooled effect (Random)	**0.336**	**[0.232, 0.432]**	**100.00**

Bold correlation coefficients indicate statistically significant effects (p < 0.05). Study weights are based on the random-effects model and are expressed as percentages. CI = confidence interval.

#### 4.3.2 Depressive symptoms

Eleven studies (*N* = 15,218) were included. The pooled correlation was *r* = 0.28 (95% CI [0.22, 0.34]). Egger’s test approached significance (*p* = 0.069). The trim-and-fill method imputed one study, adjusting the effect to *r* = 0.26 (95% CI [0.20, 0.32]). Sensitivity analysis showed a stable effect (range: *r* = 0.27 to 0.28). Detailed calculations, including conversions for studies reporting odds ratios or group means, are provided in Supplementary Table S2 (see Hammoudi et al., 2021, for an example of conversion from group means).

#### 4.3.3 Anxiety symptoms

Eight studies (*N* = 19,319) were included. The pooled correlation was *r* = 0.30 (95% CI [0.22, 0.37]). For studies reporting area under the curve (e.g., Zhou et al., 2025), correlations were estimated using established formulas. No significant publication bias was detected (Egger’s test *p* = 0.124). The trim-and-fill adjustment (one study imputed) yielded *r* = 0.28 (95% CI [0.21, 0.35]). The effect remained robust in sensitivity analyses (range: *r* = 0.28 to 0.31).

#### 4.3.4 Perceived stress

Nine studies (*N* = 12,582) were included. This analysis yielded the strongest association: *r* = 0.38 (95% CI [0.30, 0.46]). Significant funnel plot asymmetry was detected (Egger’s test *p* = 0.023). Adjusting for two imputed studies via trim-and-fill gave *r* = 0.35 (95% CI [0.27, 0.43]). The result was robust to the removal of any single study (range: *r* = 0.36 to 0.41).

### 4.4 Moderator analyses

To explore sources of heterogeneity, mixed-effects subgroup analyses and meta-regressions were conducted for outcomes with ≥10 studies (overall distress and depression).

Geographic Region was a significant moderator. Studies conducted in Asian samples showed significantly stronger associations for both overall distress (Asia: *r* = 0.38 vs. Non-Asia: *r* = 0.20; *p* = 0.042) and depression (Asia: *r* = 0.30 vs. Non-Asia: *r* = 0.16; *p* = 0.022).

Other Potential Moderators—including gender distribution (% female), specific BP measurement tool (BPS-9 vs. other), study design (cross-sectional vs. longitudinal), and risk of bias category—did not significantly moderate the effects. Similarly, meta-regressions showed that sample size and mean age were not significant continuous moderators.

These moderator results indicate that the BP-mental health association is robust across methodological variations but may be culturally contextualized, with stronger effects observed in Asian educational settings. Full moderator analysis tables are presented in Supplementary Tables S3–S10.

### 4.5 Risk of bias assessment and sensitivity analyses

#### 4.5.1 Methodological quality

**Figure 7:**
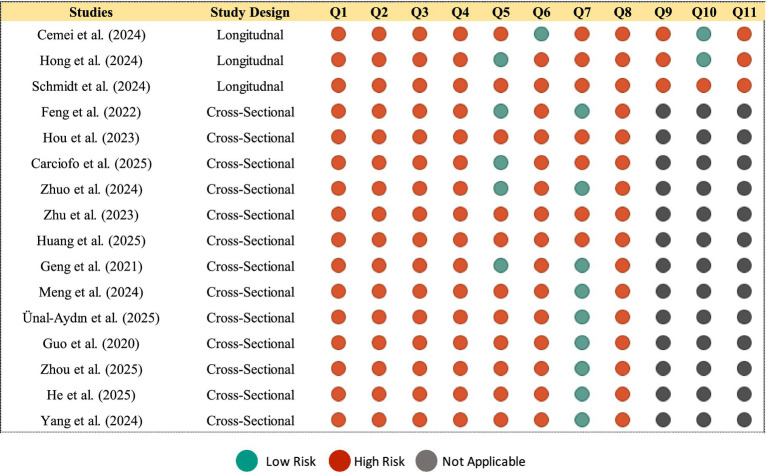
Risk of bias summary plot for included studies. Generated using the Robvis (risk of bias visualization) tool. Available in Supplementary materials.

Study quality was assessed using the Joanna Briggs Institute checklists. Of 16 studies appraised, 11 (68.8%) were rated as low risk of bias, 5 (31.2%) as moderate risk, and none as high risk. Common strengths included clear inclusion criteria and valid measurement tools. Common limitations were the use of cross-sectional designs (limiting causality), convenience sampling, and inadequate control for key confounders like academic workload. A risk-of-bias summary plot is provided in Figure 7.

#### 4.5.2 Sensitivity to study quality

To examine whether study quality influenced the findings, we compared pooled estimates from only low-risk-of-bias studies versus all studies. The differences were minimal (Δ*r* < 0.02) and non-significant, indicating that risk of bias did not substantively alter the meta-analytic conclusions.

#### 4.5.3 Comprehensive sensitivity analyses

A suite of sensitivity analyses confirmed the robustness of the primary findings:

1. Leave-One-Out Analyses: For each outcome, the pooled correlation remained within a narrow range after iteratively removing each study.
2. Model Comparison: Random-effects models were deemed appropriate given high heterogeneity; fixed-effects models produced larger but less generalizable estimates.
3. Influence Diagnostics: A few studies (e.g., Huang et al., 2025) were identified as influential due to large sample sizes or extreme effect sizes, but their removal did not change the overall interpretation.
4. Alternative Effect Size Metrics: Converting correlations to Cohen’s d yielded effects in the small-to-medium range (0.57 to 0.82), consistent with the primary interpretation.

Detailed results of all sensitivity analyses are consolidated in Supplementary Tables S11–S17.

#### 4.5.4 Narrative integration and thematic patterns

Beyond the quantitative meta-analysis, examination of the included studies reveals important thematic patterns and theoretical insights that enrich the interpretation of the meta-analytic results.

1. Bedtime Procrastination as a Transdiagnostic Marker: The consistency of BP’s association with depression, anxiety, and stress across studies frames it not as an outcome-specific behavior, but as a transdiagnostic behavioral marker of general psychological distress. This aligns with models that position BP as a concrete manifestation of broader self-regulation failure.
2. The Primacy of Stress and Coping: The strongest quantitative link with perceived stress is narratively supported by frequent references to academic pressure and daily hassles. Several studies theorized BP as a maladaptive coping mechanism—an attempt to reclaim personal time or avoid stressful thoughts through digital engagement, leading to a cycle of avoidance and sleep loss.
3. The Digital Ecosystem as an Amplifier: A prominent theme was the role of technology. Smartphones and social media were frequently cited as the primary vehicles for bedtime delay. Studies suggested that the design of digital platforms (e.g., infinite scroll, notifications) exacerbates self-regulation failure, making evening disengagement a unique modern challenge.
4. Cultural and Contextual Nuances: The moderator finding of stronger effects in Asian samples is reflected in the literature’s emphasis on high-achievement academic cultures. Studies from East Asia often explicitly linked BP to competitive academic environments and societal expectations, suggesting cultural context shapes the magnitude and meaning of the BP-distress relationship.
5. Emerging Evidence on Directionality: While causal claims are limited, the few longitudinal studies illuminated potential bidirectional pathways. For example, Cemei et al. (2024) found reciprocal relationships between BP and mental health symptoms, while Schmidt et al. (2024) demonstrated that daily stress predicted same-night BP. This supports a model of vicious cycles rather than simple linear causation.
6. Gaps and Future Directions: The narrative synthesis highlights critical gaps: a predominance of cross-sectional designs, over-reliance on self-report, and a geographic concentration in China. It points to a need for more longitudinal, experimentally oriented, and culturally diverse research that integrates objective behavioral tracking (e.g., actigraphy, digital phenotyping).

In summary, the qualitative synthesis reveals bedtime procrastination to be more than a simple sleep habit; it is a complex behavior embedded within the digital, academic, and cultural landscape of modern university life. It functions as both a consequence of distress and a potentiating factor, with digital technology serving as a key accelerant. This nuanced understanding is essential for designing effective, context-sensitive interventions.

## 5 Discussion

### 5.1 Summary of key findings

This meta-analysis of 18 studies and over 35,000 university students confirms bedtime procrastination (BP) as a significant, transdiagnostic correlate of psychological distress. More importantly, it reveals a crucial hierarchy in these associations: the link was strongest for perceived stress (*r* = 0.38), followed by anxiety (*r* = 0.30) and depressive symptoms (*r* = 0.28). This gradient, coupled with our finding that the association is significantly stronger in Asian samples than in non-Asian ones, moves the literature beyond establishing a simple correlation. It positions BP not merely as a poor sleep habit, but specifically as a stress-contingent self-regulation failure that is potentiated by cultural and academic context. The substantial heterogeneity (*I^2^* > 90%) further underscores that this relationship is not uniform but is meaningfully moderated by a broader ecosystem of individual and environmental factors.

First, we identified consistent, statistically significant positive correlations between BP and all mental health outcomes examined. This convergence across diverse studies, conducted in different countries with varied methodologies, provides robust evidence that the tendency to voluntarily delay bedtime is reliably associated with poorer mental health in the student population. The pooled effect sizes, ranging from small to moderate for depression (*r* = 0.28) to moderate for stress (*r* = 0.38), using conventional benchmarks (Cohen, 1988), are clinically meaningful. Using conventional benchmarks for correlation coefficients (Cohen, 1988), an effect size of *r* = 0.30 explains approximately 9% of the shared variance between constructs. In the context of complex, multidetermined phenomena like mental health, where numerous biological, psychological, and social factors interact, an association of this magnitude for a single, specific behavior is notable. It positions BP not as a peripheral concern, but as a substantive behavioral correlate embedded within the mental health ecology of university life.

Second, the analysis revealed a hierarchy in the strength of these associations. The relationship was strongest for perceived stress (pooled *r* = 0.38, 95% CI [0.30, 0.46]), followed by overall psychological distress (*r* = 0.34, [0.23, 0.43]), anxiety symptoms (*r* = 0.30, [0.22, 0.37]), and finally depressive symptoms (*r* = 0.28, [0.22, 0.34]). This gradient is theoretically informative. The particularly strong link with perceived stress aligns seamlessly with the *self-regulation failure* or *ego depletion* model (Baumeister et al., 1998; Kroese et al., 2016). The university environment is inherently stress-inducing, with academic demands, social transitions, and future uncertainties depleting students’ cognitive and emotional resources throughout the day. By evening, in a state of depletion, the motivation to seek immediate relief or gratification through digital entertainment or social media may overwhelm the capacity to enact the intended, long-term beneficial behavior of going to sleep. Thus, bedtime procrastination may be most directly conceptualized as a maladaptive, stress-contingent coping behavior. The slightly weaker, yet still significant, associations with depression and anxiety likely represent downstream consequences, mediated through the neurobiological and cognitive effects of the chronic sleep insufficiency that BP causes (Baglioni et al., 2011; Walker, 2009).

Third, we observed substantial and statistically significant heterogeneity across all meta-analyses (*I*^2^ values exceeding 90%). While high heterogeneity is common in meta-analyses of correlational research in behavioral sciences, it underscores a critical point: the BP-mental health link is not uniform. This variability is not merely statistical noise; it signals that other factors meaningfully moderate the strength of this relationship. Our moderator analyses indicated that geographic region was a significant factor, with studies from Asian (primarily Chinese) samples showing stronger associations than those from non-Asian contexts. This suggests the operation of cultural or contextual amplifiers, such as heightened academic pressure or specific attitudes toward sleep and productivity (Kühnel et al., 2018). The fact that other examined moderators (gender, measurement tool, study design) were not significant indicates a degree of robustness across these methodological variations. However, the large proportion of *unexplained* heterogeneity points to the influence of other unmeasured variables, such as individual differences in impulsivity or perfectionism, severity of academic workload, living conditions, or patterns of digital media use. This heterogeneity, therefore, does not undermine the main finding but rather identifies it as context-dependent, highlighting that BP’s impact is moderated by a broader ecosystem of risk and protective factors.

Fourth, the evidence base, while robust in showing association, is limited in elucidating directionality. The predominance of cross-sectional designs precludes causal inference. However, the integrated theoretical framework and the handful of available longitudinal studies suggest a dynamic, likely bidirectional or reciprocal relationship (Cemei et al., 2024; Hong et al., 2024). Psychological distress may deplete self-regulatory resources, making BP more likely. In turn, BP-induced sleep loss can exacerbate negative affect, impair emotion regulation, and heighten stress reactivity, thereby fueling further distress. This creates a vicious cycle that may entrench both poor sleep habits and psychological symptoms.

Collectively, the key findings robustly establish bedtime procrastination as a significant transdiagnostic behavioral marker associated with a spectrum of mental health difficulties in university students. Its strongest tie to perceived stress offers a clear etiological clue, framing it as a failure of self-regulation under pressure. The substantial heterogeneity reveals the context-dependent nature of this relationship, and the design limitations of the primary studies call for more sophisticated longitudinal research. Collectively, these findings make a compelling case for viewing bedtime procrastination as a legitimate and accessible target for clinical intervention and health promotion initiatives aimed at improving student well-being.

### 5.2 Theoretical integration and interpretation

#### 5.2.1 Self-regulation framework

The consistent associations observed across all mental health outcomes align with theoretical models positioning bedtime procrastination as a failure of self-regulation (Kroese et al., 2016). University students face daily cognitive and emotional demands that deplete self-regulatory resources, creating an “ego-depleted” state by evening (Baumeister et al., 1998). When in this state, the immediate gratification offered by digital entertainment and social connection disproportionately outweighs the abstract, long-term benefits of sufficient sleep. This daily self-regulation failure may establish a behavioral pattern that not only compromises sleep but also reflects broader difficulties in emotion regulation and impulse control—core deficits in both depression and anxiety disorders.

#### 5.2.2 Stress-specific mechanisms

The strongest association observed with perceived stress (*r* = 0.383) suggests bedtime procrastination may function as a maladaptive coping response to daily stressors. Rather than merely reflecting poor time management, deliberate sleep delay may represent an avoidance strategy—using engaging activities to temporarily escape academic pressures, social comparisons, or future-oriented worries. This interpretation aligns with findings that academic stressors specifically predict bedtime procrastination (Yang et al., 2024; Zhuo, 2024) and that bedtime procrastination mediates relationships between stress and poor sleep outcomes (Schmidt et al., 2024).

#### 5.2.3 The digital context: a modern amplifier

The consistent thematic identification of digital technology (e.g., smartphones, social media) as the primary vehicle for bedtime delay necessitates an update to classic self-regulation models. Our findings suggest that the university sleep environment is now a digital sleep environment. The engineered affordances of digital platforms—such as variable rewards, infinite scroll, and social notifications—exploit a state of ego depletion, making evening disengagement a uniquely modern challenge. This creates a potent feedback loop: stress depletes self-regulation → depleted individuals gravitate towards highly engaging, readily available digital rewards → engagement delays sleep → sleep loss exacerbates stress reactivity. This digital amplification hypothesis explains why BP may be particularly prevalent and damaging in contemporary student populations.

#### 5.2.4 Alternative explanations and reverse causality

While our theoretical model positions bedtime procrastination as contributing to mental health difficulties, alternative explanations warrant consideration. It is equally plausible that pre-existing depression or anxiety symptoms reduce motivation and self-regulatory capacity, making bedtime procrastination more likely. The few longitudinal studies in our synthesis suggest bidirectional relationships (Cemei et al., 2024; Schmidt et al., 2024), supporting a reciprocal rather than unidirectional model. Furthermore, third variables such as neuroticism, impulsivity, or evening chronotype could underlie both bedtime procrastination and psychological distress, representing shared vulnerability factors rather than causal pathways.

#### 5.2.5 Digital ecosystem considerations

The predominance of digital activities in bedtime procrastination behaviors (e.g., smartphone use, social media browsing) represents a novel, technology-mediated pathway to sleep disruption. Unlike traditional procrastination activities, digital platforms are engineered to sustain engagement through variable rewards and fear of missing out (FOMO), creating particular challenges for evening disengagement. This digital dimension may represent a potent, modern pathway linking behavioral procrastination to mental health, as it engages neurocognitive reward systems while simultaneously disrupting sleep–wake regulation.

In summary, our meta-analytic results support an integrated model where bedtime procrastination is best understood as a contextually amplified self-regulation failure. Daily demands, particularly stressors, deplete regulatory resources. In this depleted state, individuals in a permissive digital environment are highly susceptible to choosing immediately rewarding online activities over the deferred benefit of sleep. This choice, when repeated, leads to sleep insufficiency, which in turn impairs next-day emotion regulation and stress resilience, creating a vicious cycle. The strength of this cycle appears culturally moderated, being more potent in environments of high academic pressure.

### 5.3 Contextual and cultural considerations

#### 5.3.1 Geographic patterns

The moderator analysis revealing stronger associations in Asian samples requires careful interpretation beyond mere geographic categorization. This pattern may reflect specific cultural factors such as higher academic pressure in East Asian educational systems, collectivistic norms that amplify stress about academic performance, or differences in living arrangements (e.g., dormitory vs. family homes). Alternatively, measurement non-invariance—where BP or mental health scales function differently across cultures—could artificially inflate observed correlations. The concentration of studies in China (72.2%) limits our ability to disentangle national from cultural effects, highlighting the need for more diverse cultural sampling in future research.

#### 5.3.2 University-specific factors

The university environment presents unique vulnerabilities for bedtime procrastination development. Reduced parental oversight, flexible schedules, 24/7 digital connectivity, and high academic demands create a “perfect storm” for sleep procrastination to emerge as a coping strategy. Furthermore, the developmental timing of university years—coinciding with neural maturation of prefrontal regulatory systems—may make students particularly susceptible to establishing long-term sleep behavior patterns with lasting mental health implications.

#### 5.3.3 Gender dynamics

While gender did not emerge as a significant moderator in quantitative analyses, qualitative examination reveals important patterns. Most studies featured predominantly female samples, reflecting both recruitment patterns in psychological research and potential gender differences in help-seeking or study participation. The few studies examining gender-specific effects suggest women may be particularly vulnerable to bedtime procrastination’s mental health impacts, possibly due to differences in stress responsivity, digital media use patterns, or sleep physiology.

#### 5.3.4 Critical appraisal of the evidence base

While our meta-analysis reveals consistent associations, several methodological constraints in the primary literature warrant emphasis. First, the near-exclusive reliance on self-report measures introduces common method variance and social desirability biases. The conflation of bedtime procrastination with related constructs like insomnia severity, eveningness preference, or general procrastination in some measures may inflate observed associations. Second, the predominance of cross-sectional designs (83.3% of included studies) fundamentally limits causal inference, despite theoretical models proposing directional pathways. Third, the overrepresentation of Chinese samples and female participants raises questions about generalizability to other cultural contexts and gender groups. Fourth, few studies controlled for key confounders such as chronotype, academic workload intensity, or digital media use patterns, potentially leaving residual confounding. These limitations collectively suggest that while the BP-mental health association is robust across current studies, the evidence remains preliminary in establishing bedtime procrastination as a distinct, causal risk factor.

### 5.4 Clinical and practical implications

#### 5.4.1 Assessment as a gateway

BP, easily assessed via tools like the 9-item Bedtime Procrastination Scale (BPS), should be integrated into routine mental health and wellness screenings in university settings. It serves as a non-stigmatizing, behavioral red flag for broader psychological distress, particularly stress-related difficulties.

#### 5.4.2 Targeted intervention pathways

Our results suggest interventions should be prioritized and tailored:

1. Stress-First Approaches: Given the strongest association was with stress, primary interventions should target stress reduction (e.g., cognitive-behavioral skills for academic worry, mindfulness training) and coping resources, which may indirectly ameliorate BP.
2. Digital Hygiene Components: Any sleep intervention must explicitly address evening digital use. “Digital curfews,” app blockers, and psychoeducation on the reinforcing design of social media can be key components.
3. Contextual Adaptation: The stronger effects in Asian samples suggest interventions in high-pressure academic cultures may need to more directly address competitive stress and family expectations, rather than solely focusing on individual time management.

#### 5.4.3 University-wide policy levers

Universities have a role in shaping the environment that fosters BP. Considerations include:

1. Academic Policy: Reviewing the timing of digital assignment deadlines and promoting “assignment-free” periods to reduce 24/7 academic pressure.
2. Residence Life: Educating residential advisors to recognize and gently challenge maladaptive evening routines.
3. Health Promotion: Integrating sleep and digital wellbeing into first-year orientation and campus wellness campaigns, framing sufficient sleep as a pillar of academic success rather than a sacrifice of it.

### 5.5 Limitations

### 5.6 Methodological limitations of included studies

Our findings should be interpreted in light of several important limitations, many of which reflect constraints in the primary literature we synthesized:

- Cross-sectional predominance: The majority of included studies were cross-sectional, precluding causal inference
- Self-report bias: Reliance on retrospective self-report measures introduces common method variance and recall biases
- Sample homogeneity: Overrepresentation of Chinese students limits cross-cultural generalizability
- Measurement heterogeneity: Variations in mental health assessment tools, though all validated, may have influenced effect size estimates
- Gender imbalance: Predominantly female samples may not fully represent male students’ experiences

#### 5.6.1 Limitations of the present synthesis

- Publication bias: Despite statistical adjustments, the potential for unpublished null findings remains
- High heterogeneity: While explored through moderator analyses, substantial unexplained variance persists
- Language restriction: We included only English-language publications and used English search terms even in Chinese databases (e.g., WanFang Data), which may have excluded relevant non-English studies and introduced language bias, particularly for research from China where much work on this topic originates.
- Temporal specificity: Most studies were conducted pre- or during COVID-19, with potential pandemic-specific effects
- Construct overlap: Difficulty distinguishing bedtime procrastination from related constructs like insomnia or eveningness preference.

### 5.7 Future research directions

Our synthesis directly points to several critical avenues for future research:

#### 5.7.1 Disentangling causality and mechanism

Experimental and Micro-Longitudinal Designs: Use intensive longitudinal (e.g., daily diary) and experimental methods (e.g., resource depletion tasks) to test if daily stressors cause evening BP and to examine the real-time interplay between affect, self-control, and sleep timing.

Causal Intervention Trials: Develop and test interventions that target the primary pathways identified here (stress reduction vs. sleep hygiene vs. digital detox) to establish causal effects on BP and downstream mental health.

#### 5.7.2 Understanding context and heterogeneity

Cultural Moderators: Investigate the specific cultural, economic, or educational factors (e.g., collectivism, grade competitiveness, living arrangements) that explain the stronger BP-distress association found in Asian samples.

Digital Phenotyping: Move beyond self-report to use objective smartphone usage data to model how specific patterns of evening use (social media vs. streaming vs. gaming) relate to BP and next-day functioning.

#### 5.7.3 From correlation to application

Person-Centered Approaches: Employ latent profile analysis to identify subgroups of students with different BP patterns (e.g., “stress-procrastinators,” “reward-seekers”) for personalized intervention.

Developmental Studies: Track BP from late adolescence through university to identify critical windows for habit formation and prevention.

Scalable Solutions: Design and evaluate low-cost, scalable digital interventions (e.g., chatbot coaches, tailored notifications) that help students implement intention-behavior plans for bedtime.

#### 5.7.4 Theoretical elaboration

1. Mechanism testing: Direct examination of proposed pathways (self-regulation, stress coping, reward processing)
2. Developmental trajectories: Tracking bedtime procrastination from adolescence through adulthood
3. Digital specificity: Differentiating effects of various digital activities (social, academic, entertainment)
4. Comorbidity patterns: Understanding bedtime procrastination in relation to specific mental health disorders
5. Protective factors: Identifying resilience factors that buffer against bedtime procrastination.

#### 5.7.5 Applied research

1. Intervention development: Designing and testing targeted bedtime procrastination interventions
2. Prevention programs: Evaluating upstream approaches in secondary school transitions
3. Policy evaluation: Assessing effects of academic schedule modifications on sleep behaviors
4. Technology solutions: Developing evidence-based digital tools to support evening disengagement
5. Implementation research: Examining how to integrate sleep support into existing university services effectively

## 6 Conclusion

This systematic review and meta-analysis establishes bedtime procrastination as a significant behavioral correlate of depression, anxiety, and stress in university students. With moderate effect sizes comparable to other established risk factors, bedtime procrastination warrants attention as both a marker of psychological distress and a potential intervention target. The findings are particularly relevant given the global crisis in student mental health and growing concerns about sleep deficiency in higher education.

The results support an integrated theoretical model. In this model, bedtime procrastination emerges from daily self-regulation failures, exacerbated by digital affordances and academic stressors. It then contributes to mental health difficulties through multiple pathways, including sleep deprivation, circadian disruption, and maladaptive coping. While causal directions require further longitudinal research, the consistency and robustness of associations across diverse studies strengthen confidence in bedtime procrastination’s clinical relevance.

For university health practitioners, these findings suggest that assessing and addressing bedtime procrastination may offer a practical, non-stigmatizing entry point for supporting student mental health. For researchers, they highlight the need for more sophisticated designs that can elucidate mechanisms, directions of effect, and contextual moderators. Thus, recognizing bedtime procrastination as more than a benign habit—but rather as a behavior embedded in the complex interplay of technology, academic pressure, and mental health—may inform more effective approaches to supporting student wellbeing in the digital age.

Across 18 studies and 35,000 students, evidence converges to indicate that delaying bedtime despite the opportunity and intention to sleep may signal broader psychological distress. This distress merits attention and support. As universities worldwide grapple with rising mental health needs, bedtime procrastination offers both a warning sign and an intervention opportunity in the critical effort to support student success and wellbeing.

## Data Availability

The original contributions presented in the study are included in the article/Supplementary material, further inquiries can be directed to the corresponding author/s.

## Footnotes

1
https://www.deepseek.com
