# Supplementary material for: Bedtime procrastination and psychological distress in university students: a systematic review and meta-analysis of their association
Source: Front Psychol. 2026 Mar 5;17:1767938. doi: 10.3389/fpsyg.2026.1767938 (PMC12999920; doi:10.3389/fpsyg.2026.1767938)
Supplement: Supplementary file 1 [file Table_1.DOCX]

**Supplementary Materials for:**

***Is Bedtime Procrastination a Risk Factor for Depression, Anxiety, and Stress in University Students? A Systematic Review and Meta-Analysis***

Contents:

Supplementary Tables S1-S47

**Supplementary Table S1: Characteristics of Studies Included in the Meta-Analysis of Overall Psychological Distress**

| **Study (Author, Year)** | **Country** | **Design** | **Sample Size (N)** | **BP Measure** | **Distress Measure(s)** |
| --- | --- | --- | --- | --- | --- |
| Cemei et al. (2024) | Pakistan | Longitudinal | 683 | BPS-9 | DASS-21 (Total) |
| Feng et al. (2022) | China | Cross-sectional | 364 | BPS-9 | BDI-II |
| Hou et al. (2023) | China | Cross-sectional | 1,136 | BPS-9 | BDI-II, PSS |
| Carciofo et al. (2025) | UK | Cross-sectional | 306 | BPS-9 | DASS-21-D |
| Zhuo et al. (2024) | China | Cross-sectional | 474 | BPS-9 | Academic Workload |
| Zhu et al. (2023) | China | Cross-sectional | 668 | BPS-9 | DASS-21 (D, A, S) |
| Schmidt et al. (2024) | Germany | Daily diary | 96 | Daily BP measure | Daily Stress |
| Huang et al. (2025) | China | Cross-sectional | 5,153 | BPS-9 | PSS |
| Geng et al. (2021) | China | Cross-sectional | 355 | BPS-10 | DASS (D, A) |
| Meng et al. (2024) | China | Cross-sectional | 3,599 | BPS-9 | DASS-21-A |
| Ünal-Aydın et al. (2025) | Bosnia | Cross-sectional | 547 | BPS-9 | DASS-21 (D, A, S) |
| Guo et al. (2020) | China | Cross-sectional | 401 | BPS-9 | BDI-II |
| He et al. (2025) | China | Cross-sectional | 1,021 | BPS-9 | PSS |
| Yang et al. (2024) | China | Cross-sectional | 2,044 | BPS-9 | Academic Stress Scale |

Note.*BP = Bedtime Procrastination; BPS-9 = 9-item Bedtime Procrastination Scale; BPS-10 = 10-item version; DASS-21 = 21-item Depression, Anxiety, and Stress Scales; DASS-21-D/A/S = Depression/Anxiety/Stress subscales; BDI-II = Beck Depression Inventory-II; PSS = Perceived Stress Scale.*

**Supplementary Table S2: Complete Calculation Data for Overall Psychological Distress Meta-Analysis**

| **Study** | ***N*** | **r** | **z** | **Var(z)** | **SE(z)** | **w (FE)** | **w×z** | **w (RE)** |
| --- | --- | --- | --- | --- | --- | --- | --- | --- |
| Cemei et al. (2024) | 683 | 0.415 | 0.442 | 0.00147 | 0.0384 | 679.8 | 300.4 | 21.89 |
| Feng et al. (2022) | 364 | 0.213 | 0.216 | 0.00277 | 0.0526 | 361.0 | 78.1 | 21.29 |
| Hou et al. (2023) | 1136 | 0.385 | 0.407 | 0.00088 | 0.0297 | 1133.0 | 460.6 | 22.18 |
| ... | ... | ... | ... | ... | ... | ... | ... | ... |
| **Sum** | **20450** | – | – | – | – | **16605.0** | **8137.2** | **301.93** |

*Note.* Complete table available in supplementary materials

**Supplementary Table S3: Complete Calculation Data for Depression-Only Meta-Analysis**

| **Study** | **N** | **R** | **Z** | **Var(z)** | **w (RE)** | **Notes** |
| --- | --- | --- | --- | --- | --- | --- |
| Cemei et al. (2024) | 683 | 0.415 | 0.442 | 0.00147 | 11.23 | DASS-21 total as a proxy |
| Feng et al. (2022) | 364 | 0.213 | 0.216 | 0.00277 | 9.87 | Non-depressed subgroup |
| ... | ... | ... | ... | ... | ... | ... |
| **Sum** | **15218** | – | – | – | **100.00** |  |

Note. Full calculation table available in supplementary materials

**Supplementary Table S4: Complete Calculation Data for Anxiety-Only Meta-Analysis**

| **Study** | **N** | **r** | **z** | **Var(z)** | **w (RE)** | **Notes** |
| --- | --- | --- | --- | --- | --- | --- |
| Cemei et al. (2024) | 683 | 0.415 | 0.442 | 0.00147 | 15.43 | DASS-21 total as a proxy |
| Zhu et al. (2023) | 668 | 0.190 | 0.192 | 0.00150 | 15.21 | Direct correlation |
| Hong et al. (2024) | 1423 | 0.280 | 0.288 | 0.00070 | 15.89 | Converted from OR |
| Geng et al. (2021) | 355 | 0.460 | 0.497 | 0.00284 | 15.05 | Direct correlation |
| Meng et al. (2024) | 3599 | 0.334 | 0.347 | 0.00028 | 16.12 | Direct correlation |
| Hammoudi et al. (2021) | 591 | 0.195 | 0.198 | 0.00170 | 15.38 | Calculated from means/SDs |
| Ünal-Aydın et al. (2025) | 547 | 0.187 | 0.189 | 0.00184 | 15.32 | Direct correlation |
| Zhou et al. (2025) | 6543 | 0.310 | 0.321 | 0.00015 | 16.60 | Estimated from AUC |
| **Sum** | **19319** | – | – | – | **100.00** |  |

Note. Full calculation table available in supplementary materials.

**Supplementary Table S5: Complete Calculation Data for Stress-Only Meta-Analysis**

| **Study** | **N** | **r** | **z** | **Var(z)** | **w (RE)** | **Notes** |
| --- | --- | --- | --- | --- | --- | --- |
| Cemei et al. (2024) | 683 | 0.415 | 0.442 | 0.00147 | 14.21 | DASS-21 total as proxy |
| Hou et al. (2023) | 1136 | 0.477 | 0.518 | 0.00088 | 14.89 | Direct correlation |
| Zhu et al. (2023) | 668 | 0.260 | 0.266 | 0.00150 | 14.05 | Direct correlation |
| Schmidt et al. (2024) | 96 | 0.090 | 0.090 | 0.01075 | 11.23 | Daily stress measure |
| Huang et al. (2025) | 5153 | 0.620 | 0.725 | 0.00019 | 15.34 | Direct correlation |
| He et al. (2025) | 1021 | 0.320 | 0.332 | 0.00098 | 14.62 | Direct correlation |
| Yang et al. (2024) | 2044 | 0.540 | 0.604 | 0.00049 | 15.18 | Direct correlation |
| Ünal-Aydın et al. (2025) | 547 | 0.266 | 0.273 | 0.00184 | 14.12 | Direct correlation |
| Zhuo et al. (2024) | 474 | 0.300 | 0.310 | 0.00212 | 13.36 | Academic workload as proxy |
| **Sum** | **12582** | – | – | – | **100.00** |  |

Note. Full calculation table available in supplementary materials.

**Supplementary Table S6: Characteristics of Studies Included in the Meta-Analysis of Depressive Symptoms**

| **Study (Author, Year)** | **Country** | **Design** | **Sample Size (N)** | **BP Measure** | **Depression Measure** |
| --- | --- | --- | --- | --- | --- |
| Cemei et al. (2024) | Pakistan | Longitudinal | 683 | BPS-9 | DASS-21-D* |
| Feng et al. (2022) | China | Cross-sectional | 364 | BPS-9 | BDI-II |
| Hou et al. (2023) | China | Cross-sectional | 1,136 | BPS-9 | BDI-II |
| Carciofo et al. (2025) | UK | Cross-sectional | 306 | BPS-9 | DASS-21-D |
| Zhu et al. (2023) | China | Cross-sectional | 668 | BPS-9 | DASS-21-D |
| Geng et al. (2021) | China | Cross-sectional | 355 | BPS-10 | DASS-D |
| Guo et al. (2020) | China | Cross-sectional | 401 | BPS-9 | BDI-II |
| Hong et al. (2024) | China | Longitudinal | 1,423 | SPS-8 | DASS-21-D |
| Hammoudi et al. (2021) | Lebanon | Cross-sectional | 591 | BPS-9 | PHQ-9 |
| Ünal-Aydın et al. (2025) | Bosnia | Cross-sectional | 547 | BPS-9 | DASS-21-D |
| Zhou et al. (2025) | China | Cross-sectional | 6,543 | BPS-8 | DASS-21-D |
| Zhu et al. (2023)† | China | Cross-sectional | 668 | BPS-9 | DASS-21-D |

Note. BP = Bedtime Procrastination; BPS-9 = 9-item Bedtime Procrastination Scale; BPS-10 = 10-item version; SPS-8 = 8-item Sleep Procrastination Scale; DASS-21-D = Depression subscale of the 21-item Depression, Anxiety, and Stress Scales; BDI-II = Beck Depression Inventory-II; PHQ-9 = Patient Health Questionnaire-9. *Cemei et al. (2024) used DASS-21 total score as a proxy for depression due to unavailable subscale data. †Zhu et al. (2023) appears twice—once in the overall and once here for depression-specific correlation; correlation is from the depression subscale.

**Supplementary Table S7: Random-Effects Meta-Analysis of Bedtime Procrastination and Depressive Symptoms**

| **Study** | **r** | **95% CI** | **Weight (%)** |
| --- | --- | --- | --- |
| Cemei et al. (2024) | 0.415 | [0.340, 0.485] | 11.23 |
| Feng et al. (2022) | 0.213 | [0.110, 0.312] | 9.87 |
| Hou et al. (2023) | 0.292 | [0.237, 0.345] | 11.52 |
| Carciofo et al. (2025) | 0.206 | [0.097, 0.311] | 9.74 |
| Zhu et al. (2023) | 0.210 | [0.134, 0.284] | 10.98 |
| Geng et al. (2021) | 0.470 | [0.403, 0.533] | 10.65 |
| Guo et al. (2020) | 0.233 | [0.135, 0.327] | 10.15 |
| Hong et al. (2024)* | 0.280† | [0.230, 0.328] | 11.41 |
| Hammoudi et al. (2021)‡ | 0.185 | [0.105, 0.263] | 10.81 |
| Ünal-Aydın et al. (2025) | 0.134 | [0.052, 0.215] | 10.69 |
| Zhou et al. (2025)§ | 0.310 | [0.290, 0.330] | 12.01 |
| **Pooled Effect (Random)** | **0.277** | **[0.218, 0.335]** | **100.00** |

Note. CI = confidence interval. *Correlation derived from DASS-21 total score. †Correlation converted from odds ratio (Chinn, 2000). ‡Correlation calculated from group means and standard deviations. §Correlation estimated from area under the curve.

**Supplementary Table S8: Characteristics of Studies Included in the Meta-Analysis of Anxiety Symptoms**

| **Study (Author, Year)** | **Country** | **Design** | **Sample Size (N)** | **BP Measure** | **Anxiety Measure** |
| --- | --- | --- | --- | --- | --- |
| Cemei et al. (2024) | Pakistan | Longitudinal | 683 | BPS-9 | DASS-21-A* |
| Zhu et al. (2023) | China | Cross-sectional | 668 | BPS-9 | DASS-21-A |
| Hong et al. (2024) | China | Longitudinal | 1,423 | SPS-8 | DASS-21-A |
| Geng et al. (2021) | China | Cross-sectional | 355 | BPS-10 | DASS-A |
| Meng et al. (2024) | China | Cross-sectional | 3,599 | BPS-9 | DASS-21-A |
| Hammoudi et al. (2021) | Lebanon | Cross-sectional | 591 | BPS-9 | GAD-7 |
| Ünal-Aydın et al. (2025) | Bosnia | Cross-sectional | 547 | BPS-9 | DASS-21-A |
| Zhou et al. (2025) | China | Cross-sectional | 6,543 | BPS-8 | DASS-21-A |
| Feng et al. (2022)† | China | Cross-sectional | 364 | BPS-9 | – |
| Schmidt et al. (2024)‡ | Germany | Daily diary | 96 | Daily BP measure | – |

Note. BP = Bedtime Procrastination; BPS-9 = 9-item Bedtime Procrastination Scale; BPS-10 = 10-item version; SPS-8 = 8-item Sleep Procrastination Scale; DASS-21-A = Anxiety subscale of the 21-item Depression, Anxiety, and Stress Scales; GAD-7 = Generalized Anxiety Disorder-7. *Cemei et al. (2024) used DASS-21 total score as a proxy for anxiety due to unavailable subscale data. †Feng et al. (2022) did not measure anxiety; excluded from analysis. ‡Schmidt et al. (2024) did not measure anxiety; excluded from analysis.

**Supplementary Table S9: Random-Effects Meta-Analysis of Bedtime Procrastination and Anxiety Symptoms**

| **Study** | **R** | **95% CI** | **Weight (%)** |
| --- | --- | --- | --- |
| Cemei et al. (2024)* | 0.415 | [0.340, 0.485] | 15.43 |
| Zhu et al. (2023) | 0.190 | [0.113, 0.265] | 15.21 |
| Hong et al. (2024)† | 0.280 | [0.230, 0.328] | 15.89 |
| Geng et al. (2021) | 0.460 | [0.393, 0.523] | 15.05 |
| Meng et al. (2024) | 0.334 | [0.301, 0.366] | 16.12 |
| Hammoudi et al. (2021)‡ | 0.195 | [0.115, 0.273] | 15.38 |
| Ünal-Aydın et al. (2025) | 0.187 | [0.105, 0.267] | 15.32 |
| Zhou et al. (2025)§ | 0.310 | [0.290, 0.330] | 16.60 |
| **Pooled Effect (Random)** | **0.295** | **[0.221, 0.367]** | **100.00** |

Note. CI = confidence interval. *Correlation derived from DASS-21 total score. †Correlation converted from odds ratio (Chinn, 2000). ‡Correlation calculated from group means and standard deviations. §Correlation estimated from area under the curve.

**Supplementary Table S10: Characteristics of Studies Included in the Meta-Analysis of Perceived Stress**

| **Study (Author, Year)** | **Country** | **Design** | **Sample Size (N)** | **BP Measure** | **Stress Measure** |
| --- | --- | --- | --- | --- | --- |
| Cemei et al. (2024) | Pakistan | Longitudinal | 683 | BPS-9 | DASS-21-S* |
| Hou et al. (2023) | China | Cross-sectional | 1,136 | BPS-9 | PSS |
| Zhu et al. (2023) | China | Cross-sectional | 668 | BPS-9 | DASS-21-S |
| Schmidt et al. (2024) | Germany | Daily diary | 96 | Daily BP measure | Daily Stress |
| Huang et al. (2025) | China | Cross-sectional | 5,153 | BPS-9 | PSS |
| He et al. (2025) | China | Cross-sectional | 1,021 | BPS-9 | PSS |
| Yang et al. (2024) | China | Cross-sectional | 2,044 | BPS-9 | Academic Stress Scale |
| Ünal-Aydın et al. (2025) | Bosnia | Cross-sectional | 547 | BPS-9 | DASS-21-S |
| Zhuo et al. (2024)† | China | Cross-sectional | 474 | BPS-9 | Academic Workload |

Note. BP = Bedtime Procrastination; BPS-9 = 9-item Bedtime Procrastination Scale; DASS-21-S = Stress subscale of the 21-item Depression, Anxiety, and Stress Scales; PSS = Perceived Stress Scale. *Cemei et al. (2024) used DASS-21 total score as a proxy for stress due to unavailable subscale data. †Zhuo et al. (2024) used Academic Workload as a stress proxy.

**Supplementary Table S11: Random-Effects Meta-Analysis of Bedtime Procrastination and Perceived Stress**

| **Study** | **r** | **95% CI** | **Weight (%)** |
| --- | --- | --- | --- |
| Cemei et al. (2024)* | 0.415 | [0.340, 0.485] | 14.21 |
| Hou et al. (2023) | 0.477 | [0.425, 0.526] | 14.89 |
| Zhu et al. (2023) | 0.260 | [0.185, 0.333] | 14.05 |
| Schmidt et al. (2024) | 0.090 | [-0.113, 0.287] | 11.23 |
| Huang et al. (2025) | 0.620 | [0.594, 0.645] | 15.34 |
| He et al. (2025) | 0.320 | [0.259, 0.379] | 14.62 |
| Yang et al. (2024) | 0.540 | [0.496, 0.582] | 15.18 |
| Ünal-Aydın et al. (2025) | 0.266 | [0.185, 0.344] | 14.12 |
| Zhuo et al. (2024)† | 0.300 | [0.210, 0.386] | 13.36 |
| **Pooled Effect (Random)** | **0.383** | **[0.298, 0.463]** | **100.00** |

Note. CI = confidence interval; weights based on random-effects model. *Cemei et al. (2024): used DASS-21 total score as proxy. †Zhuo et al. (2024): used Academic Workload as stress proxy.

**Supplementary Table S12: Summary of All Meta-Analyses Results**

| **Outcome** | **k** | **N** | **Pooled r [95% CI]** | **I² (%)** | **τ²** |
| --- | --- | --- | --- | --- | --- |
| Overall Distress | 14 | 20,450 | 0.336 [0.232, 0.432] | 97.9 | 0.044 |
| Depression | 11 | 15,218 | 0.277 [0.218, 0.335] | 94.7 | 0.012 |
| Anxiety | 8 | 19,319 | 0.295 [0.221, 0.367] | 95.5 | 0.014 |
| Stress | 9 | 12,582 | 0.383 [0.298, 0.463] | 98.4 | 0.027 |

Note. All pooled correlations were statistically significant (p < .001).

**Supplementary Table S13: Summary of Random-Effects Meta-Analyses of Bedtime Procrastination and Mental Health Outcomes in University Students**

| **Outcome** | **Studies (k)** | **Total N** | **Pooled r [95% CI]** | **p-value** | **I² [95% CI]** | **τ² [95% CI]** |
| --- | --- | --- | --- | --- | --- | --- |
| **Overall Psychological Distress** | 14 | 20,450 | 0.336 [0.232, 0.432] | < .001 | 97.9% [97.3, 98.4] | 0.044 [0.021, 0.098] |
| **Depressive Symptoms** | 11 | 15,218 | 0.277 [0.218, 0.335] | < .001 | 94.7% [92.5, 96.3] | 0.012 [0.006, 0.028] |
| **Anxiety Symptoms** | 8 | 19,319 | 0.295 [0.221, 0.367] | < .001 | 95.5% [93.8, 96.8] | 0.014 [0.007, 0.033] |
| **Perceived Stress** | 9 | 12,582 | 0.383 [0.298, 0.463] | < .001 | 98.4% [98.0, 98.7] | 0.027 [0.013, 0.061] |

Note. r = Pearson correlation coefficient; CI = confidence interval; I² = heterogeneity index; τ² = between-study variance.

**Supplementary Table S14: Subgroup Analysis for Overall Psychological Distress by Geographic Region**

| **Subgroup** | **k** | **Pooled r [95% CI]** | **I² (%)** | **τ²** | **Between-Group Q** | **p** |
| --- | --- | --- | --- | --- | --- | --- |
| Asia | 11 | 0.381 [0.305, 0.453] | 97.8 | 0.032 | 4.12 | .042 |
| Non-Asia | 3 | 0.202 [0.021, 0.374] | 94.5 | 0.028 |  |  |

**Supplementary Table S15: Subgroup Analysis for Overall Psychological Distress by BP Measurement Tool**

| **Subgroup** | **k** | **Pooled r [95% CI]** | **I² (%)** | **τ²** | **Between-Group Q** | **p** |
| --- | --- | --- | --- | --- | --- | --- |
| BPS-9 | 12 | 0.351 [0.245, 0.450] | 98.1 | 0.045 | 0.87 | .351 |
| Other | 2 | 0.285 [0.130, 0.430] | 92.3 | 0.012 |  |  |

**Supplementary Table S16: Subgroup Analysis for Overall Psychological Distress by Study Design**

| **Subgroup** | **k** | **Pooled r [95% CI]** | **I² (%)** | **τ²** | **Between-Group Q** | **p** |
| --- | --- | --- | --- | --- | --- | --- |
| Cross-sectional | 13 | 0.345 [0.238, 0.445] | 98.0 | 0.046 | 0.33 | .565 |
| Longitudinal | 1 | 0.415 [0.340, 0.485] | – | – |  |  |

**Supplementary Table S17: Subgroup Analysis for Overall Psychological Distress by Risk of Bias**

| **Subgroup** | **k** | **Pooled r [95% CI]** | **I² (%)** | **τ²** | **Between-Group Q** | **P** |
| --- | --- | --- | --- | --- | --- | --- |
| Low RoB (JBI ≥ 7) | 8 | 0.328 [0.210, 0.438] | 98.3 | 0.049 | 0.15 | .699 |
| Moderate/High RoB (JBI < 7) | 6 | 0.345 [0.185, 0.489] | 97.2 | 0.037 |  |  |

**Supplementary Table S18: Meta-Regression Results for Overall Psychological Distress**

| **Moderator** | **Coefficient (β)** | **SE** | **95% CI** | **z** | **p** | **R² (%)** |
| --- | --- | --- | --- | --- | --- | --- |
| % Female | 0.0021 | 0.0015 | [-0.0008, 0.0050] | 1.40 | .162 | 12.4 |
| Mean Age | -0.021 | 0.018 | [-0.056, 0.014] | -1.17 | .242 | 9.8 |
| Sample Size (log) | -0.045 | 0.038 | [-0.120, 0.030] | -1.18 | .238 | 10.1 |

**Supplementary Table S19: Subgroup Analysis for Depression by Geographic Region**

| **Subgroup** | **k** | **Pooled r [95% CI]** | **I² (%)** | **τ²** | **Between-Group Q** | **p** |
| --- | --- | --- | --- | --- | --- | --- |
| Asia | 9 | 0.298 [0.242, 0.352] | 93.5 | 0.010 | 5.23 | .022 |
| Non-Asia | 2 | 0.156 [0.045, 0.263] | 85.2 | 0.008 |  |  |

**Supplementary Table S20: Subgroup Analysis for Depression by BP Measurement Tool**

| **Subgroup** | **k** | **Pooled r [95% CI]** | **I² (%)** | **τ²** | **Between-Group Q** | **p** |
| --- | --- | --- | --- | --- | --- | --- |
| BPS-9 | 9 | 0.269 [0.203, 0.333] | 95.1 | 0.013 | 1.89 | .169 |
| Other | 2 | 0.328 [0.245, 0.407] | 87.3 | 0.006 |  |  |

**Supplementary Table S21: Subgroup Analysis for Depression by Study Design**

| **Subgroup** | **k** | **Pooled r [95% CI]** | **I² (%)** | **τ²** | **Between-Group Q** | **p** |
| --- | --- | --- | --- | --- | --- | --- |
| Cross-sectional | 10 | 0.273 [0.212, 0.332] | 94.9 | 0.012 | 0.44 | .507 |
| Longitudinal | 1 | 0.280 [0.230, 0.328] | – | – |  |  |

**Supplementary Table S22: Subgroup Analysis for Depression by Risk of Bias**

| **Subgroup** | **k** | **Pooled r [95% CI]** | **I² (%)** | **τ²** | **Between-Group Q** | **p** |
| --- | --- | --- | --- | --- | --- | --- |
| Low RoB (JBI ≥ 7) | 7 | 0.261 [0.192, 0.327] | 95.3 | 0.013 | 1.02 | .312 |
| Moderate/High RoB (JBI < 7) | 4 | 0.305 [0.215, 0.390] | 93.8 | 0.011 |  |  |

**Supplementary Table S23: Meta-Regression Results for Depression**

| **Moderator** | **Coefficient (β)** | **SE** | **95% CI** | **z** | **p** | **R² (%)** |
| --- | --- | --- | --- | --- | --- | --- |
| % Female | 0.0018 | 0.0012 | [-0.0006, 0.0042] | 1.50 | .134 | 18.7 |
| Mean Age | -0.015 | 0.012 | [-0.039, 0.009] | -1.25 | .211 | 15.3 |
| Sample Size (log) | -0.028 | 0.025 | [-0.077, 0.021] | -1.12 | .263 | 11.9 |

Note. None of the continuous moderators were statistically significant.

**Supplementary Table S24: Sensitivity Analysis Comparing Low Risk-of-Bias vs. All Studies**

| **Outcome** | **Analysis** | **k** | **Pooled r [95% CI]** | **I² (%)** | **Δ from Main Analysis*** |
| --- | --- | --- | --- | --- | --- |
| **Overall Psychological Distress** |  |  |  |  |  |
|  | Main analysis (all studies) | 14 | 0.336 [0.232, 0.432] | 97.9 | – |
|  | Low RoB only | 8 | 0.328 [0.210, 0.438] | 98.3 | −0.008 |
| **Depressive Symptoms** |  |  |  |  |  |
|  | Main analysis (all studies) | 11 | 0.277 [0.218, 0.335] | 94.7 | – |
|  | Low RoB only | 7 | 0.261 [0.192, 0.327] | 95.3 | −0.016 |
| Outcome | Analysis | k | Pooled r [95% CI] | I² (%) | Difference |
| Overall Distress | Low RoB only | 8 | 0.328 [0.210, 0.438] | 98.3 | -0.008 |
|  | All studies | 14 | 0.336 [0.232, 0.432] | 97.9 | (reference) |
| Depression | Low RoB only | 7 | 0.261 [0.192, 0.327] | 95.3 | -0.016 |
|  | All studies | 11 | 0.277 [0.218, 0.335] | 94.7 | (reference) |

Note. Δ = difference in pooled correlation coefficient (r) compared to the main analysis. RoB = risk of bias.

**Supplementary Table S25: Leave-One-Out Analysis for Overall Psychological Distress**

| **Study Omitted** | **k** | **Pooled r** | **95% CI** | **Change from Full** | **Most Influential Rank** |
| --- | --- | --- | --- | --- | --- |
| **Full Analysis** | 14 | **0.336** | **[0.232, 0.432]** | – | – |
| Cemei et al. (2024) | 13 | 0.331 | [0.226, 0.429] | -0.005 | 5 |
| Feng et al. (2022) | 13 | 0.345 | [0.241, 0.443] | +0.009 | 7 |
| Hou et al. (2023) | 13 | 0.332 | [0.227, 0.430] | -0.004 | 4 |
| Carciofo et al. (2025) | 13 | 0.343 | [0.238, 0.441] | +0.007 | 6 |
| Zhuo et al. (2024) | 13 | 0.339 | [0.234, 0.437] | +0.003 | 3 |
| Zhu et al. (2023) | 13 | 0.343 | [0.238, 0.441] | +0.007 | 6 |
| Schmidt et al. (2024) | 13 | 0.351 | [0.245, 0.449] | **+0.015** | **2** |
| Huang et al. (2025) | 13 | 0.319 | [0.214, 0.417] | **-0.017** | **1** |
| Geng et al. (2021) | 13 | 0.329 | [0.224, 0.427] | -0.007 | 8 |
| Meng et al. (2024) | 13 | 0.337 | [0.231, 0.435] | +0.001 | 9 |
| Ünal-Aydın et al. (2025) | 13 | 0.343 | [0.238, 0.441] | +0.007 | 6 |
| Guo et al. (2020) | 13 | 0.341 | [0.236, 0.439] | +0.005 | 4 |
| He et al. (2025) | 13 | 0.338 | [0.233, 0.436] | +0.002 | 2 |
| Yang et al. (2024) | 13 | 0.323 | [0.218, 0.421] | -0.013 | 3 |

**Supplementary Table S26: Leave-One-Out Analysis for Depression**

| **Study Omitted** | **k** | **Pooled r** | **95% CI** | **Change from Full** | **Most Influential Rank** |
| --- | --- | --- | --- | --- | --- |
| **Full Analysis** | 11 | **0.277** | **[0.218, 0.335]** | – | – |
| Cemei et al. (2024) | 10 | 0.265 | [0.205, 0.323] | -0.012 | 3 |
| Feng et al. (2022) | 10 | 0.282 | [0.222, 0.340] | +0.005 | 6 |
| Hou et al. (2023) | 10 | 0.275 | [0.215, 0.333] | -0.002 | 5 |
| Carciofo et al. (2025) | 10 | 0.283 | [0.223, 0.341] | +0.006 | 7 |
| Zhu et al. (2023) | 10 | 0.282 | [0.222, 0.340] | +0.005 | 6 |
| Geng et al. (2021) | 10 | 0.265 | [0.205, 0.323] | **-0.012** | **3** |
| Guo et al. (2020) | 10 | 0.280 | [0.220, 0.338] | +0.003 | 4 |
| Hong et al. (2024) | 10 | 0.276 | [0.216, 0.334] | -0.001 | 2 |
| Hammoudi et al. (2021) | 10 | 0.281 | [0.221, 0.339] | +0.004 | 5 |
| Ünal-Aydın et al. (2025) | 10 | 0.285 | [0.225, 0.343] | **+0.008** | **1** |
| Zhou et al. (2025) | 10 | 0.274 | [0.214, 0.332] | -0.003 | 4 |

**Supplementary Table S27: Leave-One-Out Analysis for Anxiety**

| **Study Omitted** | **k** | **Pooled r** | **95% CI** | **Change from Full** |
| --- | --- | --- | --- | --- |
| **Full Analysis** | 8 | **0.295** | **[0.221, 0.367]** | – |
| Cemei et al. (2024) | 7 | 0.279 | [0.203, 0.352] | -0.016 |
| Zhu et al. (2023) | 7 | 0.308 | [0.233, 0.380] | +0.013 |
| Hong et al. (2024) | 7 | 0.298 | [0.223, 0.371] | +0.003 |
| Geng et al. (2021) | 7 | 0.279 | [0.203, 0.352] | **-0.016** |
| Meng et al. (2024) | 7 | 0.287 | [0.211, 0.360] | -0.008 |
| Hammoudi et al. (2021) | 7 | 0.301 | [0.226, 0.373] | +0.006 |
| Ünal-Aydın et al. (2025) | 7 | 0.302 | [0.227, 0.374] | +0.007 |
| Zhou et al. (2025) | 7 | 0.291 | [0.215, 0.364] | -0.004 |

**Supplementary Table S28: Leave-One-Out Analysis for Stress**

| **Study Omitted** | **k** | **Pooled r** | **95% CI** | **Change from Full** | **Most Influential** |
| --- | --- | --- | --- | --- | --- |
| **Full Analysis** | 9 | **0.383** | **[0.298, 0.463]** | – | – |
| Cemei et al. (2024) | 8 | 0.378 | [0.291, 0.460] | -0.005 |  |
| Hou et al. (2023) | 8 | 0.375 | [0.288, 0.457] | -0.008 |  |
| Zhu et al. (2023) | 8 | 0.394 | [0.307, 0.475] | +0.011 |  |
| Schmidt et al. (2024) | 8 | 0.408 | [0.321, 0.489] | **+0.025** | **Yes** |
| Huang et al. (2025) | 8 | 0.361 | [0.274, 0.443] | **-0.022** | **Yes** |
| He et al. (2025) | 8 | 0.390 | [0.303, 0.471] | +0.007 |  |
| Yang et al. (2024) | 8 | 0.370 | [0.283, 0.452] | -0.013 |  |
| Ünal-Aydın et al. (2025) | 8 | 0.391 | [0.304, 0.472] | +0.008 |  |
| Zhuo et al. (2024) | 8 | 0.392 | [0.305, 0.473] | +0.009 |  |

**Supplementary Table S29: Model Comparison for All Outcomes**

| **Outcome** | **Model** | **k** | **Pooled r** | **95% CI** | **I² (%)** | **τ²** | **Difference** |
| --- | --- | --- | --- | --- | --- | --- | --- |
| Overall | Random | 14 | 0.336 | [0.232, 0.432] | 97.9 | 0.044 | Reference |
|  | Fixed | 14 | 0.415 | [0.403, 0.427] | – | – | +0.079 |
| Depression | Random | 11 | 0.277 | [0.218, 0.335] | 94.7 | 0.012 | Reference |
|  | Fixed | 11 | 0.312 | [0.300, 0.324] | – | – | +0.035 |
| Anxiety | Random | 8 | 0.295 | [0.221, 0.367] | 95.5 | 0.014 | Reference |
|  | Fixed | 8 | 0.334 | [0.321, 0.347] | – | – | +0.039 |
| Stress | Random | 9 | 0.383 | [0.298, 0.463] | 98.4 | 0.027 | Reference |
|  | Fixed | 9 | 0.477 | [0.464, 0.490] | – | – | +0.094 |

**Supplementary Table S30: Studies with Cook's Distance > 0.5 (Influential)**

| **Outcome** | **Study** | **Cook's D** | **DFBETAS (Intercept)** | **Influential?** |
| --- | --- | --- | --- | --- |
| Overall | Huang et al. (2025) | 0.82 | 1.23 | Yes |
| Overall | Schmidt et al. (2024) | 0.61 | -0.98 | Yes |
| Depression | Geng et al. (2021) | 0.58 | 1.12 | Yes |
| Depression | Ünal-Aydın et al. (2025) | 0.53 | -0.89 | Yes |
| Stress | Huang et al. (2025) | 0.91 | 1.45 | Yes |
| Stress | Schmidt et al. (2024) | 0.67 | -1.02 | Yes |
| Anxiety | None | <0.5 | – | No |

**Supplementary Table S31: Studies Contributing Most to Heterogeneity (τ²)**

| **Outcome** | **Study** | **% Contribution to Q** | **Residual I² if Removed** |
| --- | --- | --- | --- |
| Overall | Huang et al. (2025) | 45.9% | 97.5% |
| Overall | Meng et al. (2024) | 11.9% | 97.8% |
| Depression | Zhou et al. (2025) | 28.3% | 93.1% |
| Depression | Geng et al. (2021) | 25.1% | 93.5% |
| Stress | Huang et al. (2025) | 51.2% | 98.0% |
| Stress | Yang et al. (2024) | 24.6% | 98.1% |

**Supplementary Table S32: Pooled Effects in Cohen's d Metric**

| **Outcome** | **k** | **r** | **d** | **95% CI for d** | **Interpretation*** |
| --- | --- | --- | --- | --- | --- |
| Overall | 14 | 0.336 | 0.717 | [0.474, 0.967] | Medium |
| Depression | 11 | 0.277 | 0.573 | [0.447, 0.702] | Small-Medium |
| Anxiety | 8 | 0.295 | 0.620 | [0.452, 0.793] | Medium |
| Stress | 9 | 0.383 | 0.822 | [0.617, 1.033] | Medium-Large |

**Cohen's d guidelines: 0.2 = small, 0.5 = medium, 0.8 = large*

**Supplementary Table S33: Back-Transformation Consistency Check**

| **Outcome** | **Pooled z** | **Back-transformed r** | **Original r** | **Difference** |
| --- | --- | --- | --- | --- |
| Overall | 0.3496 | 0.336 | 0.336 | 0.000 |
| Depression | 0.2845 | 0.277 | 0.277 | 0.000 |
| Anxiety | 0.3042 | 0.295 | 0.295 | 0.000 |
| Stress | 0.4034 | 0.383 | 0.383 | 0.000 |

**Supplementary Table S34: Sensitivity by Region (Asian vs. Non-Asian)**

| **Outcome** | **Region** | **k** | **Pooled r** | **95% CI** | **Difference from Full** |
| --- | --- | --- | --- | --- | --- |
| Overall | Asia only | 11 | 0.381 | [0.305, 0.453] | +0.045 |
|  | Non-Asia only | 3 | 0.202 | [0.021, 0.374] | -0.134 |
| Depression | Asia only | 9 | 0.298 | [0.242, 0.352] | +0.021 |
|  | Non-Asia only | 2 | 0.156 | [0.045, 0.263] | -0.121 |

**Supplementary Table S35: Sensitivity by BP Scale**

| **Outcome** | **Scale** | **k** | **Pooled r** | **95% CI** |
| --- | --- | --- | --- | --- |
| Overall | BPS-9 only | 12 | 0.351 | [0.245, 0.450] |
|  | Other scales | 2 | 0.285 | [0.130, 0.430] |
| Depression | BPS-9 only | 9 | 0.269 | [0.203, 0.333] |
|  | Other scales | 2 | 0.328 | [0.245, 0.407] |

**Supplementary Table S36: Overall Sample Characteristics Across All Included Studies (k = 18)**

| **Characteristic** | **Total (N = 35,097)*** | **Mean per Study** | **Range Across Studies** | **Studies Reporting (k)** |
| --- | --- | --- | --- | --- |
| **Total Participants** | 35,097 | 1,950 | 96 – 6,543 | 18 |
| **Mean Age** | – | 19.8 years | 18.8 – 22.2 years | 15 |
| **Age Range** | – | 17–30 years | 17–23 to 18–51 | 12 |
| **Female Percentage** | 65.4% | 65.4% | 18.8% – 86.6% | 17 |
| **Male Percentage** | 34.6% | 34.6% | 13.4% – 81.2% | 17 |

**Total N differs from meta-analysis N due to different study inclusions*

**Supplementary Table S37: Geographic Distribution of Studies and Samples**

| **Region** | **Studies (k)** | **Percentage** | **Total Participants** | **Mean Sample Size** |
| --- | --- | --- | --- | --- |
| China | 13 | 72.2% | 27,763 | 2,136 |
| Other Asia (Pakistan, Lebanon) | 2 | 11.1% | 1,274 | 637 |
| Europe (UK, Germany, Bosnia) | 3 | 16.7% | 6,060 | 2,020 |
| **Total** | **18** | **100%** | **35,097** | **1,950** |

**Supplementary Table S38: Study Design Characteristics**

| **Design** | **Studies (k)** | **Percentage** | **Total Participants** | **Mean Sample Size** |
| --- | --- | --- | --- | --- |
| Cross-sectional | 15 | 83.3% | 28,138 | 1,876 |
| Longitudinal (2-wave) | 2 | 11.1% | 2,106 | 1,053 |
| Daily diary | 1 | 5.6% | 96 | 96 |
| **Total** | **18** | **100%** | **30,340†** | **1,686** |

*†Daily diary participant count differs as N = days (1,320) not individuals*

**Supplementary Table S39: Bedtime Procrastination Measurement Characteristics**

| **Measure** | **Studies (k)** | **Percentage** | **Items** | **Response Scale** | **Mean Cronbach's α** | **Range of α** |
| --- | --- | --- | --- | --- | --- | --- |
| BPS-9 (9-item) | 14 | 77.8% | 9 | 5-point Likert | 0.85 | 0.74 – 0.96 |
| BPS-10 (10-item) | 1 | 5.6% | 10 | 5-point Likert | 0.80 | – |
| SPS-8 (Sleep Procrastination) | 1 | 5.6% | 8 | 5-point Likert | 0.91 | – |
| Daily diary measure | 1 | 5.6% | Single item | Minutes | – | – |
| Unspecified/adapted | 1 | 5.6% | Varies | Varies | 0.86 | – |
| **Total** | **18** | **100%** |  |  | **0.85** | **0.74 – 0.96** |

**Supplementary Table S40: Mental Health Measurement Characteristics**

| **Construct** | **Primary Measure** | **Studies (k)** | **Percentage** | **Mean Cronbach's α** | **Range of α** |
| --- | --- | --- | --- | --- | --- |
| Depression | DASS-21 Depression | 7 | 38.9% | 0.88 | 0.85 – 0.94 |
|  | BDI-II | 3 | 16.7% | 0.89 | 0.87 – 0.91 |
|  | PHQ-9 | 1 | 5.6% | 0.86 | – |
|  | Various/combined | 7 | 38.9% | 0.84 | 0.78 – 0.92 |
| **Depression Total** |  | **18** | **100%** | **0.87** | **0.78 – 0.94** |
| Anxiety | DASS-21 Anxiety | 6 | 33.3% | 0.86 | 0.82 – 0.91 |
|  | GAD-7 | 1 | 5.6% | 0.89 | – |
|  | Various/combined | 3 | 16.7% | 0.84 | 0.80 – 0.88 |
|  | Not measured | 8 | 44.4% | – | – |
| **Anxiety Total** |  | **10** | **55.6%** | **0.86** | **0.80 – 0.91** |
| Stress | PSS | 3 | 16.7% | 0.86 | 0.84 – 0.88 |
|  | DASS-21 Stress | 3 | 16.7% | 0.87 | 0.85 – 0.90 |
|  | Academic Stress Scales | 3 | 16.7% | 0.85 | 0.82 – 0.88 |
|  | Daily Stress | 1 | 5.6% | 0.79 | – |
|  | Various/combined | 2 | 11.1% | 0.83 | 0.80 – 0.86 |
|  | Not measured | 6 | 33.3% | – | – |
| **Stress Total** |  | **12** | **66.7%** | **0.84** | **0.79 – 0.90** |

**Supplementary Table S41: Bedtime Procrastination Descriptive Statistics**

| **Study** | **N** | **Mean** | **SD** | **Possible Range** | **Actual Range** | **Scale Type** |
| --- | --- | --- | --- | --- | --- | --- |
| Cemei et al. (2024) | 683 | 19.83 | 7.01 | 9–45 | 9–45 | Sum (9 items) |
| Feng et al. (2022) | 364 | 23.74 | 6.19 | 9–45 | 9–45 | Sum (9 items) |
| Hou et al. (2023) | 1,136 | 2.91 | 0.80 | 1–5 | 1–5 | Item average |
| Carciofo et al. (2025) | 306 | 32.47 | 6.79 | 9–45 | 9–45 | Sum (9 items) |
| Zhu et al. (2023) | 668 | 3.35 | 0.66 | 1–5 | 1–5 | Item average |
| Huang et al. (2025) | 5,153 | 24.38 | 7.42 | 9–45 | 9–45 | Sum (9 items) |
| Geng et al. (2021) | 355 | 25.37 | 6.15 | 10–50 | 10–50 | Sum (10 items) |
| **Weighted Average** | **8,665** | **21.5** | **6.4** | **–** | **–** | **–** |

Note:*Different scoring methods prevent direct averaging; weighted by sample size where comparable*

**Supplementary Table S42: Mental Health Symptom Descriptive Statistics**

| **Measure** | **Studies (k)** | **Weighted Mean** | **Weighted SD** | **Clinical Cut-off*** | **% Above Cut-off†** |
| --- | --- | --- | --- | --- | --- |
| **Depression** |  |  |  |  |  |
| BDI-II (0–63) | 3 | 8.9 | 9.5 | ≥14 | 26.9% |
| DASS-21 Depression (0–21) | 7 | 5.2 | 4.8 | ≥10 | 31.4% |
| PHQ-9 (0–27) | 1 | 11.9 | 6.3 | ≥10 | 58.7% |
| **Anxiety** |  |  |  |  |  |
| DASS-21 Anxiety (0–21) | 6 | 5.8 | 4.9 | ≥8 | 42.3% |
| GAD-7 (0–21) | 1 | 10.3 | 5.8 | ≥10 | 51.2% |
| **Stress** |  |  |  |  |  |
| PSS (0–40) | 3 | 38.2 | 7.9 | ≥20 | 92.1% |
| DASS-21 Stress (0–21) | 3 | 6.9 | 4.6 | ≥15 | 18.7% |

**Common clinical cut-offs for each measure. †Estimated from study-reported prevalence or calculated from means/SDs assuming normal distribution.*

**Supplementary Table S43: Reliability Coefficients (Cronbach's α) Synthesis**

| **Measure** | **Studies (k)** | **Mean α** | **SD of α** | **Minimum** | **Maximum** | **% ≥ .70** | **% ≥ .80** |
| --- | --- | --- | --- | --- | --- | --- | --- |
| **BP Measures** |  |  |  |  |  |  |  |
| BPS-9 | 14 | 0.85 | 0.06 | 0.74 | 0.96 | 100% | 85.7% |
| All BP measures | 16 | 0.84 | 0.07 | 0.74 | 0.96 | 100% | 81.3% |
| **Depression Measures** |  |  |  |  |  |  |  |
| DASS-21 Depression | 7 | 0.88 | 0.03 | 0.85 | 0.94 | 100% | 100% |
| BDI-II | 3 | 0.89 | 0.02 | 0.87 | 0.91 | 100% | 100% |
| All depression | 13 | 0.87 | 0.04 | 0.78 | 0.94 | 100% | 100% |
| **Anxiety Measures** |  |  |  |  |  |  |  |
| DASS-21 Anxiety | 6 | 0.86 | 0.03 | 0.82 | 0.91 | 100% | 100% |
| All anxiety | 9 | 0.85 | 0.04 | 0.79 | 0.91 | 100% | 88.9% |
| **Stress Measures** |  |  |  |  |  |  |  |
| PSS | 3 | 0.86 | 0.02 | 0.84 | 0.88 | 100% | 100% |
| DASS-21 Stress | 3 | 0.87 | 0.03 | 0.85 | 0.90 | 100% | 100% |
| All stress | 8 | 0.84 | 0.04 | 0.79 | 0.90 | 100% | 87.5% |

**Supplementary Table S44: Temporal Stability of Measures**

| **Study** | **Measure** | **Time Interval** | **Test-Retest r** | **N** |
| --- | --- | --- | --- | --- |
| Cemei et al. (2024) | BPS | 6 months | 0.68 | 683 |
|  | DASS-21 Total | 6 months | 0.72 | 683 |
| Hong et al. (2024) | SPS | 6 months | 0.71 | 1,423 |
|  | DASS-21 Subscales | 6 months | 0.65–0.69 | 1,423 |
| Schmidt et al. (2024) | Daily BP | 14 days (ICC) | 0.42 | 96 |
| **Weighted Average** | **–** | **–** | **0.67** | **2,202** |

**Supplementary Table S45: Correlations Among Study-Level Variables (k = 18)**

| **Variable** | **1** | **2** | **3** | **4** | **5** | **6** |
| --- | --- | --- | --- | --- | --- | --- |
| 1. Sample Size (log) | 1 |  |  |  |  |  |
| 2. % Female | -0.18 | 1 |  |  |  |  |
| 3. Mean Age | -0.32 | 0.14 | 1 |  |  |  |
| 4. BP Reliability (α) | 0.25 | -0.21 | 0.08 | 1 |  |  |
| 5. Effect Size (r) | -.29* | 0.12 | -0.19 | 0.17 | 1 |  |
| 6. Publication Year | .41* | -0.09 | -0.28 | 0.33 | -0.14 | 1 |

**p < .05*

**Supplementary Table S46: Completeness of Reported Data**

| **Data Element** | **Studies Reporting (k)** | **Percentage** | **Notes** |
| --- | --- | --- | --- |
| Sample size | 18 | 100% | All studies reported |
| Gender distribution | 17 | 94.4% | 1 study NR |
| Mean age | 15 | 83.3% | 3 studies NR |
| Age range | 12 | 66.7% | 6 studies NR |
| BP mean and SD | 16 | 88.9% | 2 studies NR/incomplete |
| BP reliability (α) | 16 | 88.9% | 2 studies NR |
| Mental health means/SDs | 14 | 77.8% | 4 studies incomplete |
| Correlation coefficients | 14 | 77.8% | 4 studies required conversion |
| **Overall Completeness** | **–** | **84.7%** | **Weighted average** |

**Supplementary Table S47: Missing Data Handling in Primary Studies**

| **Approach** | **Studies (k)** | **Percentage** | **Examples** |
| --- | --- | --- | --- |
| Complete case analysis | 12 | 66.7% | Excluded incomplete responses |
| Not reported | 4 | 22.2% | Unclear handling |
| Imputation mentioned | 2 | 11.1% | Mean substitution, regression |
| **Total** | **18** | **100%** |  |
