# Supplementary material for: Bedtime procrastination and psychological distress in university students: a systematic review and meta-analysis of their association
Source: Front Psychol. 2026 Mar 5;17:1767938. doi: 10.3389/fpsyg.2026.1767938 (PMC12999920; doi:10.3389/fpsyg.2026.1767938)
Supplement: Supplementary file 2 [file Table_2.DOCX]

**Supplementary Materials for:**

***Is Bedtime Procrastination a Risk Factor for Depression, Anxiety, and Stress in University Students? A Systematic Review and Meta-Analysis***

Contents:

Supplementary Figures S1-S4

**Supplementary Figure S1. Funnel plot of correlation coefficients (r) between bedtime procrastination and overall psychological distress in university students. Each point represents a study; the vertical dashed line indicates the pooled effect size.**

**
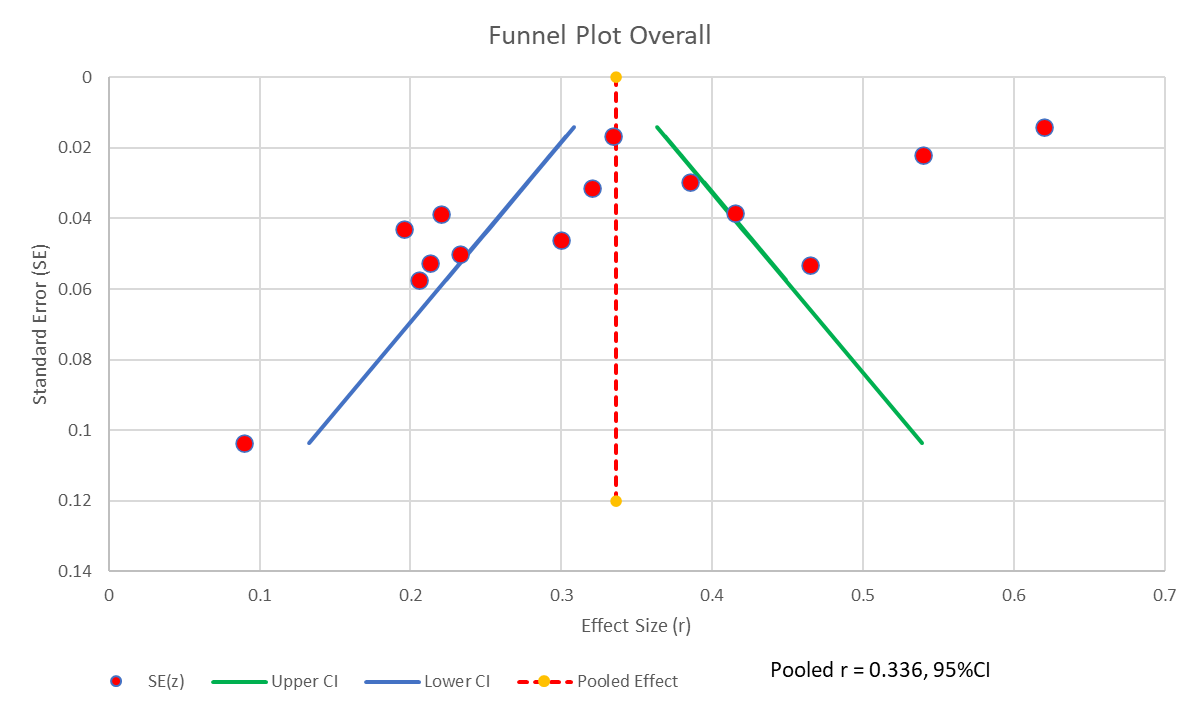
**

***Note: Visual inspection suggests a slight asymmetry in the lower-left quadrant, indicating possible publication bias. Egger’s regression test was significant (t = 3.12, p = .008).***

**Supplementary Figure S2.** **Funnel plot of correlation coefficients (r) between bedtime procrastination and depressive symptoms in university students.**


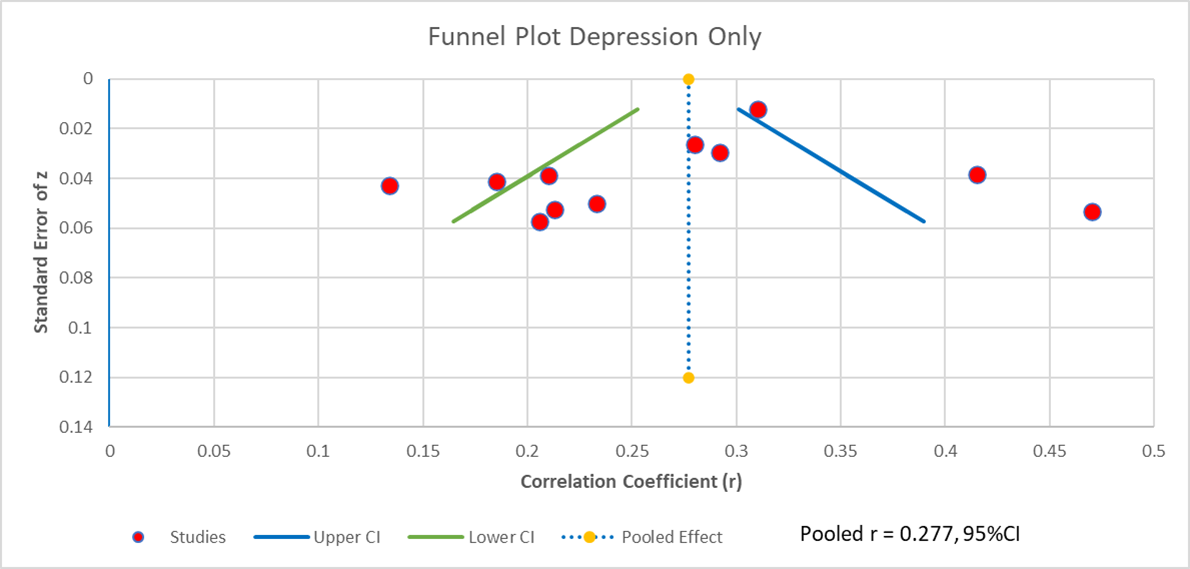


***Note:*** *Mild asymmetry is observed. Egger’s test approached significance (t = 2.04, p = .069). The trim-and-fill method imputed one missing study.*

**Supplementary Figure S3. Funnel plot of correlation coefficients (r) between bedtime procrastination and anxiety symptoms in university students.**

**
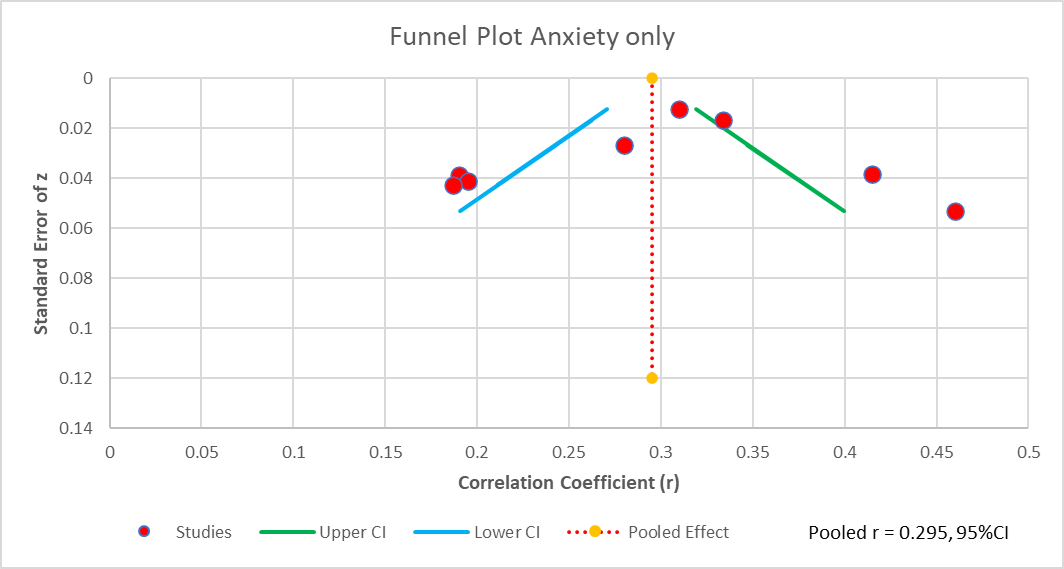
**

***Note: Mild asymmetry is present. Egger’s test was not statistically significant (t = 1.78, p = .124). One study was imputed using the trim-and-fill method.***

**Supplementary Figure S4.** **Funnel plot of correlation coefficients (r) between bedtime procrastination and perceived stress in university students**.


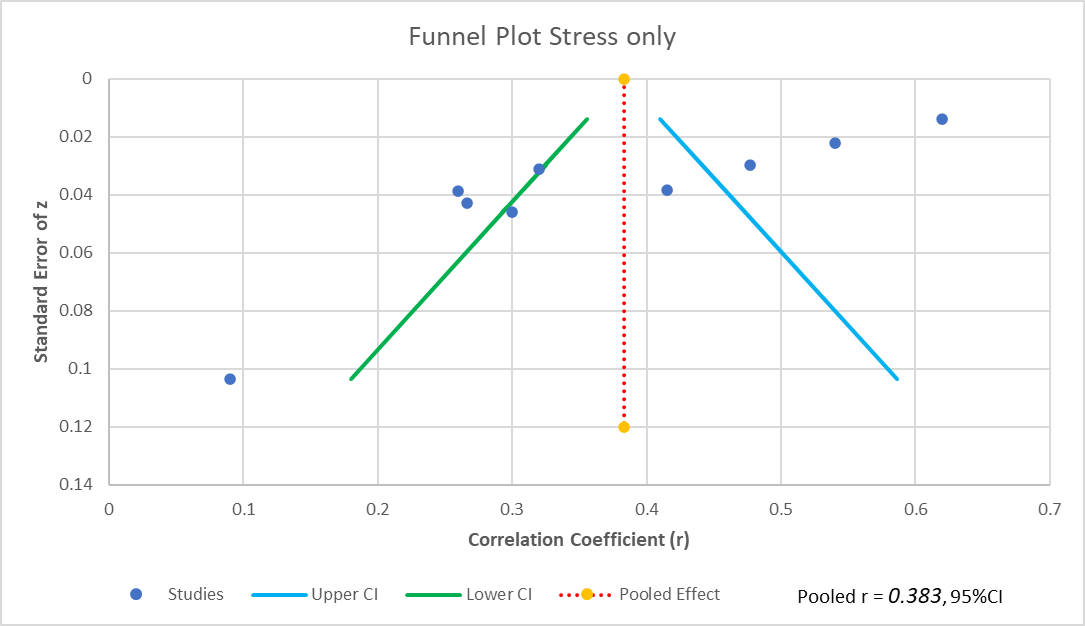


***Note:*** *Asymmetry is detected, particularly in the lower-left quadrant. Egger’s test was significant (t = 2.89, p = .023). Two studies were imputed using the trim-and-fill method.*
